# Federated learning for privacy-preserving skin cancer classification using deep neural networks

**DOI:** 10.3389/fonc.2026.1720748

**Published:** 2026-04-27

**Authors:** Mohammed A. M. Alfalahi, Oğuz Karan, Sefer Kurnaz, Ayça Kurnaz Türkben

**Affiliations:** 1 Electrical and Computer Engineering Department, Engineering College, Altinbas University, Istanbul, Türkiye; 2 College of Management and Economics, Al Iraqia University, Baghdad, Iraq; 3 Siemens Digital Industries Foundational Technologies, Istanbul, Türkiye; 4 Faculty of Engineering and Natural Sciences, Department, Engineering College, Rumeli University, Istanbul, Türkiye

**Keywords:** federated learning, skin cancer classification, medical imaging, deep learning, MobileNetV2, privacy preservation

## Abstract

**Introduction:**

Skin cancer is a significant health issue in the entire world and there is a need to have diagnostic systems that are precise, enlargeable and privacy safeguarding. The heterogeneity of the institution and the issue of patient confidentiality frequently restrict the use of centralized deep learning in the medical imaging field because of data-sharing limitations and heterogeneity across different institutions. The solution is federation learning (FL), which allows joint training of models without transfer of unprocessed clinical data.

**Methods:**

In this work, it is suggested to implement a privacy-sensitive FL architecture in skin cancer classification on the basis of decentralized clinical environments. The framework has compared two aggregation topologies, centralized Federated Averaging (FedAvg) and a decentralized ring-based peer-to-peer strategy on five simulated non-IID clinical client data distributions. In order to capture the institutional heterogeneity, we constructed a heterogeneity sensitive client partitioning algorithm that is measured by KullbackLeibler divergence. The suggested framework was also compared to standard FL algorithms, such as FedProx and SCAFFOLD, and was evaluated at various non-IID levels of severity. The backbone architectures implemented were MobileNetV2 and VGG16 and tested on two benchmark data sets, which were ISIC and Skin Cancer: Malignant vs. Benign. It was reported that the performance is measured in several random seeds and has 95% confidence intervals, and the balance of privacy and utility is measured using the settings of differential privacy.

**Results:**

MobileNetV2 with the ring-based FL topology was the best performing of all the considered configurations. This setting achieved an accuracy of 98.88% (95% CI: 97.92–99.84%) and F1-score of 98.80% which is significantly better than the centralized baseline (p < 0.01). In both datasets, MobileNetV2 has been able to achieve higher convergence rates, reduced communication overhead, and greater performance in terms of resistance to heterogeneous client distributions, compared to VGG16 in federation. The degradation of performance with increasing severity of non-IID was gradual, which validates that the planned framework is scalable and robust.

**Discussion:**

The results show that federated learning is able to be as effective as or even more effective than centralized learning in skin cancer classification without sacrificing patient privacy. The MobileNetV2 performance is higher, implying that lightweight architectures are specifically suited in FL due to their effective gradient propagation and low communication cost. Altogether, the suggested framework will be a feasible and regulation-conformed solution of AI-aided dermatological diagnosis in distributed healthcare settings.

## 1 Introduction

Skin cancer is among the most prevalent cancers globally, where millions of new cases are diagnosed each year, which have immense impact on population health outcomes of people across the world (1–3). The related morbidity highlights the high urgency of timely and accurate diagnostic treatment measures to minimize deaths and improve treatment effectiveness (4, 5). Historically, clinical diagnosis has predominantly relied on expert dermatological assessments, which are limited by the subjective nature of visual perception, inter-observer variability, and restricted availability in disadvantaged areas (6, 7).

The latest development in the field of machine learning (ML) and artificial intelligence (AI) has already demonstrated much potential in improving dermatological diagnosis due to these limitations, ushering in a new era of earlier diagnosis and personalized treatment plans (8–13).

Melanoma is one of the most common subtypes of skin cancer that happens to be the most deadly type of skin cancer (14). World Health Organization (WHO) epidemiological data of 2017 showed that approximately 132,000 melanoma cases and approximately 2–3 million non-melanoma cases are identified every year in the world (15, 16). More recent estimates report 331,722 new skin cancer cases identified in 2022, according to (17, 18). Key risk factors contributing to the disease burden include excessive ultraviolet (UV) radiation exposure, frequent sunburns, tanning bed usage, and hereditary predispositions (19). Melanoma is regarded as especially frequent among those who have light skin, and it is the most frequent cause of death in skin cancer cases.

The vast majority of currently available diagnostic tools are based on unimodal sources of data, i.e., structured tabular data, clinical text notation, or dermoscopic images, which restricts diagnostic quality and completeness (20–24). Such a silo method can result in misclassification or a lack of comprehensive clinical understanding. Thus, current research recommends a multimodal framework including different types of data to enhance the contextualization and the diagnostic fidelity (10, 25–28). Multimodal models have the potential to achieve a much higher accuracy, interpretability, and robustness in skin cancer classification problems due to their ability to incorporate heterogeneous information, such as dermoscopic images, patient demographics, as well as molecular or genomic profiles (29–31).

Multimodal research has been given an additional impetus by the release of new high-quality multimodal AI models such as ChatGPT-4.0 by OpenAI in March 2023, which has stimulated further growth in the use of multimodal AI systems in a broader range of medical AI applications (29, 32). In this respect, the convolutional neural networks or CNNs have proven to be extremely skilled at the task of skin lesion classification. Nonetheless, this model training requires very large datasets of labels, which are usually distributed among various hospitals and research facilities. In many cases, these datasets cannot be consolidated in central repositories due to strict governmental data privacy regulations, such as the General Data Protection Regulation (GDPR), as well as institutional policies that limit data sharing (33).

The solution to this dilemma is the emergence of federated learning (FL). FL was initially introduced by Google and is a decentralized mechanism of training models that enables the use of multiple nodes and the maintenance of sensitive patient data as both local and private information (34). With this paradigm shift, the hospitals can create strong diagnostic models collaboratively, without actually sharing raw data with each other. FL has demonstrated significant potential in the medical field, especially regarding privacy-sensitive tasks, such as skin cancer diagnosis, to encourage strong generalization and ensure full adherence to regulatory policies (29, 35–39).

The paper gives a detailed federated learning architecture of privacy-sensitive skin cancer classification, which fills in the vital gaps existing in the body of literature on realistic federated learning applications to medical imaging. In contrast to previous studies in which simplified FL configurations are used, our model deals with clinical heterogeneity in practice by using two or more institutions with different data features. The main contributions are:

- Heterogeneity-Aware Federated Architecture: Our new client partitioning plan is based on the explicit modelling of institutional data heterogeneity, quantitatively. Data with controlled non-IID characteristics, namely different ratios of classes (40:60 to 60:40 benign-to-malignant) and different preprocessing pipelines, are used to simulate inter-institutional variability in each of the five simulated clinical clients. We present the application of the Kullback-Leibler (KL) divergence to measure and report the level of heterogeneity among the clients.
- Multi-Topology FL Comparison with State-of-the-Art Baseline: We implement and rigorously compare four FL strategies: (i) FedAvg (centralized aggregation), (ii) Ring AllReduce (decentralized peer-to-peer), (iii) FedProx (proximal regularization for heterogeneous settings), and (iv) SCAFFOLD (variance reduction via control variates). This is the major comparison of the limitations of the previous studies that have only tested single FL algorithms.
- Privacy Analysis and Differential Privacy Integration: Our formal privacy guarantees are differentiated privacy analysis and are reported privacy budget (ϵ) at various noise scales. We study possible vectors of data leakage in augmentation pipelines, and apply such protective measures as local-only augmentation and gradient clipping.
- Scalability Analysis Across Client Configurations: To show the achievement of scalability, we test system performance using 3, 5, and 7 clients, and we test the convergence behavior as well as the efficiency of communication, and the pattern of accuracy degradation as the federation size is varied.
- Theoretical Analysis of Architecture Suitability: We offer the theoretical explanation of why MobileNetV2 performs better in federated environments, which compares the distributions of gradient magnitudes, the communication cost per round, and the convergence rate guarantees. This fills the gap in the literature that exists on the selection of architectures in FL.
- Rigorous Statistical Validation with Confidence Intervals: All experiments are performed on five random seeds, and their results are given as the means ± of those with the standard deviation and 95% confidence interval. Our statistical significance tests are paired t-tests, and Wilcoxon signed-rank tests, and effect sizes (Cohen’s d) are provided in comparisons.
- Non-IID Severity Analysis: We also non-systematically assess model robustness with skewness of label distributions on three levels of non-IID severity (mild, moderate, severe) that can give some information about FL performance under conditions of different degrees of data heterogeneity.

## 2 Related work

Federated Learning (FL) and its multimodal extension (MMFL) have become a considerable part of the healthcare industry due to their inherent ability to address two key concerns, namely, inter-institutional heterogeneity and data privacy. In this case, we provide an overview and position recent research in the area of deep learning-based diagnosis of skin cancer, such as (i) deep learning-based image analysis of skin lesions, (ii) the evolution of multimodal federated learning in medical images, and (iii) how mechanisms of knowledge transfer can be incorporated into federated systems.

### 2.1 Advancements in skin lesion analysis

There has been great progress in the use of deep learning methods to classify, segment, and predict malignancy in skin lesions. Architectures such as ResNet, EfficientNet, and DenseNet have been extensively utilized to achieve high performance in both binary and multiclass skin cancer detection applications (20, 40–43). Other recent papers have also included multimodal data (e.g., dermoscopic images, clinical features (e.g., age, gender, lesion location), patient history) to improve classification accuracy and minimize model uncertainty (5, 42, 44–46).

Nevertheless, the models usually use massive, centrally managed datasets, which are usually unrealistic in terms of data governance in a healthcare facility. Elementary needs of centralized data aggregation are not only causing privacy issues, but also making it difficult to generalize across different institutional distributions. This encourages the use of federated learning methods to enable joint training of a model on data that does not need to be shared directly.

### 2.2 Multimodal federated learning in medical imaging

The paradigm of federated learning has gained recent popularity as a privacy protection method, particularly in sensitive areas such as healthcare. Although there is a smaller body of research on unimodal FL, in which clients process a single form of data (i.e., images), the multimodal federated learning (MMFL) extension is relatively new and complicated to study (47–49). The goal of MMFL frameworks is to harness the heterogeneity of the data sources dispersed among clients to form more multi-dimensional diagnostic models.

Luceri et al. (48) introduced a multimodal paradigm of explainability in computer-aided skin cancer diagnosis with emphasis on interpretability even in situations of wrongful categorization. In yet another study, an FL system that is collaborative was presented that allows learning inter-institutional data in a secure manner without compromising patient privacy (50). Moreover, individualized FL strategies have been proposed to make model behavior adapt to local distributions of data, which improves clinical performance (51).

New developments in personal federated learning of medical images have shown exceptional improvement. Chen et al. (52) introduced a patient-specific federated denoising framework for low-dose CT that leverages large language models (LLMs) to jointly encode patient anatomy and scanning physics, thereby improving denoising performance while better preserving individual anatomical details. Equally, Yang et al. (53) proposed a physics-based personalized FL hypernetwork model of CT imaging that learns to dynamically generate client-specific model parameters in response to the local data characteristics, which proves the possibility of physics-informed personalization in medical imaging FL.

It is worth noting that recent studies have extensively explored the integration of artificial intelligence and deep learning within federated learning frameworks, particularly in healthcare and dermatological applications. Sumaiya and Ali (54) presented a comprehensive survey on federated learning-assisted deep learning methods for skin cancer detection, highlighting key challenges and future research directions. Malik et al. (55) discussed the integration of artificial intelligence in healthcare systems and its role in enhancing disease detection and diagnostic accuracy. Furthermore, Sashidharavi et al. (56) proposed an efficient approach for skin disease classification using multi-dataset fusion and attention mechanisms to improve model performance.Moreover, Palit et al. (57) emphasized the significance of artificial intelligence in advancing medical diagnostics and innovation. Kurtansky et al. (58) introduced the large-scale SLICE-3D dataset, which provides extensive skin lesion image crops to support the development of robust diagnostic models. In addition, Borazjani et al. (59) investigated multi-modal federated learning for cancer staging under non-IID data conditions, demonstrating improved performance in heterogeneous environments. Alhafiz and Bashuhail (60) further analyzed the impact of non-IID medical imaging data within federated learning frameworks, particularly in the context of COVID-19 datasets.

Most MMFL systems deployed nowadays, however, model that every data modality is available to every client, which is hardly true in a real-world deployment. This creates the problem of the missing modality challenge (29, 47, 50) whereby some clients use incomplete or unbalanced input data. Mechanisms of mitigation against this have been synthetic modality generation, data imputation, and cross-modal alignment.

Expanding the scope of FL and MMFL to more complex tasks like MRI reconstruction and synthesis has also been done in recent works (61–63), and shows that they are useful in more general medical imaging applications. It is worth noting that recent studies have examined the implementation of deep learning within federated models with specialized dermatological applications in particular (64–69). Arangasamy et al. (64) came up with a privacy-preserving model to classify skin diseases using an attention mechanism and federated learning. Khan et al. (65) showed that ensemble deep learning methods are useful in distributed locations to segment skin lesions. Moreover, recent full-scale surveys (66, 67) also note the possibility of a combination of FL and advanced deep learning structures to achieve better outcomes in diagnostics in the field of dermatology.

Federated deep convolutional neural network architectures have demonstrated good results in skin cancer diagnosis in relation to dermatology (70), but most studies have not been thoroughly evaluated at diverse FL architectures and heterogeneity. Federated transfer learning in skin cancer was studied by Naz et al. (68), and Alhafiz et al. (69) investigated the resilience of FL systems to a wide range of non-IID data distributions in medical imaging.

Nevertheless, the current FL models of skin lesion analysis have their shortcomings in a few areas: (i) incomplete comparison of multiple FL algorithms (FedAvg, FedProx, SCAFFOLD), (ii) inadequate analysis of non-IID severity effects, (iii) no privacy guarantees and unlike analysis of differential privacy, and (iv) a suboptimal evaluation of scalability. The paper fills these gaps by presenting a detailed federated learning model, which has been experimentally tested on a multidimensional basis.

### 2.3 Federated learning’s knowledge transfer

Knowledge transfer is crucial in federated learning, especially when clients have partial modalities or diverse data distributions. One promising approach involves knowledge distillation, where a global or ensemble model transfers soft predictions or feature representations to local models without direct data access.

Knowledge distillation was used by Islam et al. (44) to create a lightweight, highly effective skin cancer classifier that was trained in a federated fashion. Other studies have explored self-supervised and semi-supervised distillation techniques to enhance generalization in low-resource clients (71). Furthermore, recent investigations (72, 73) report the successful integration of knowledge distillation into skin lesion classifiers, resulting in competitive performance despite limitations in local data. A summary of the related federated and multimodal learning literature for skin cancer detection is provided in **Table 1**.

**Table 1:** The overview of the available literature on federated learning and multimodal learning in the detection of skin cancer.

Ref. & YoP	Short summary	Task	Included modalities	FL type	Dataset
Luceri et al. (2022) (48)	Multimodal explainable FL framework for skin cancer with interpretable outputs.	Binary Classification	Dermoscopic + Clinical Metadata	MMFL	ISIC Archive
Islam et al. (2022) (44)	Lightweight classifier trained using knowledge distillation in FL setting.	Binary Classification	Dermoscopic Images	FL + Knowledge Transfer	HAM10000
Qayyum et al. (2022) (50)	Collaborative FL across hospitals with privacy-preserving aggregation.	Medical Imaging	Imaging + Text	Standard FL	Institutional Datasets
Ref. & YoP	Short Summary	Task	Included Modalities	FL Type	Dataset
Agbley et al. (2021) (47)	FL with missing modalities using synthetic modality estimation.	General Diagnosis	Heterogeneous Modalities	MMFL (Incomplete)	Custom Data
Wang et al. (2023) (71)	Self-supervised distillation method for lesion classification in FL.	Binary Classification	Dermoscopic Images	FL + KD + SSL	ISIC 2018
Qin et al. (2023) (61)	FL for MRI synthesis and reconstruction with privacy guarantees.	MRI Reconstruction	MRI + Genomics	MMFL + Personalized FL	BraTS
Al Rakhami et al. (2024) (70)	DCNN-based FL framework for skin cancer without data sharing.	Binary Classification	Dermoscopic Images	FedAvg	ISIC 2019
Huang et al. (2024) (29)	Review of MMFL challenges: heterogeneity, missing modalities.	Survey/Review	Imaging + Text + Metadata	MMFL/Personalized FL	Not Applicable

## 3 Dataset overview

### 3.1 Skin cancer: malignant vs. benign dataset

The much-narrowed version of the ISIC Archive, the dataset of Skin Cancer: Malignant vs. Benign was created to differentiate between benign and malignant skin lesions in binary classification exercises. It has been extensively employed in the creation and assessment of machine learning models for the detection of skin cancer.

Dataset Composition

The dataset consists of two balanced classes:

- Benign: 1,800 dermoscopic images of non-cancerous moles.
- Malignant: 1,800 dermoscopic images of cancerous skin lesions.

Every image has a resolution of roughly 224 × 224 pixels and is supplied in RGB format. The dataset is organized into two separate directories—one for each class—making it straightforward to load and preprocess for binary classification pipelines.

Source and Licensing

This dataset is derived from the publicly accessible ISIC Archive, a global repository of dermatological images. All associated rights are retained by the ISIC Archive, and users are expected to comply with its terms of use. The dataset provider has explicitly stated that no derivative models trained on this data should be monetized.

Usage in This Study

This dataset is used in our study as the standard by which different federated learning and centralized models are evaluated in a controlled binary classification environment. Its balanced distribution class-wise renders it applicable in the evaluation of the model performance in terms of accuracy, F1-score, precision, recall, and AUC.

**Figure 1:**
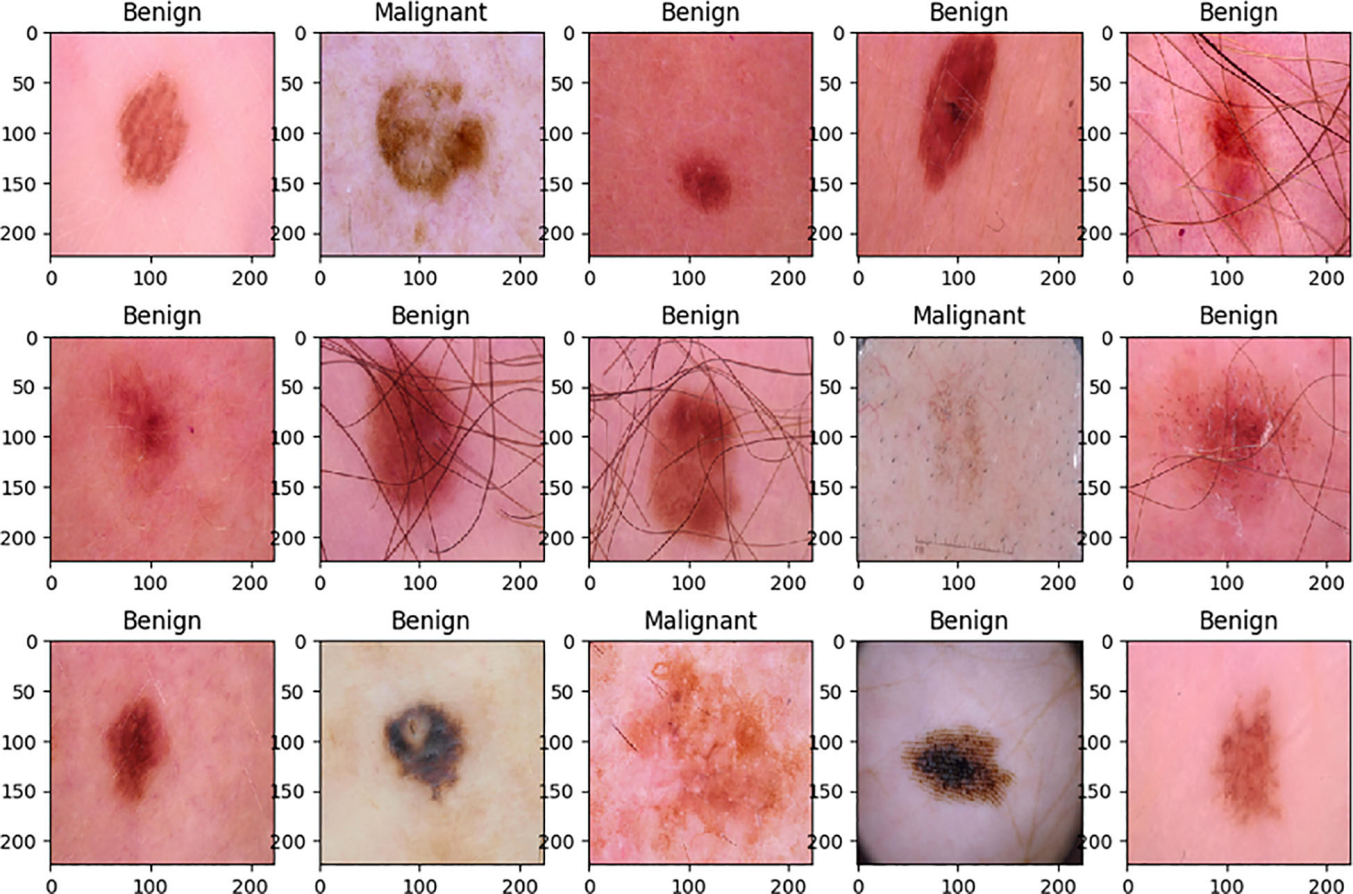
The dataset of skin cancer: malignant vs. benign has sample dermoscopic images of benign and malignant lesions in different visual features such as color, shape, texture, and the occurrence of artifacts such as hair or lighting reflections.

**Figure 1** illustrates a selection of dermoscopic images sampled from both benign and malignant classes. The diversity in appearance—including pigmentation, border irregularities, and surrounding artifacts—highlights the challenge of accurate visual classification and motivates the use of automated deep learning approaches.

### 3.2 Skin Cancer ISIC dataset

The Skin Cancer ISIC dataset is a multiclass dermoscopic image dataset that consists of 2357 images obtained by the International Skin Imaging Collaboration (ISIC). The data set allows categorizing a wide variety of dermatological diseases and is aimed at assisting in the work of multi-classification of skin lesions. It reflects real-world clinical variability and diagnostic complexity, making it a valuable benchmark for deep learning research.

#### 3.2.1 Dataset composition

All images were pre-sorted according to the ISIC taxonomy and organized into the following nine distinct classes:

- Actinic Keratosis (AK)
- Basal Cell Carcinoma (BCC)
- Dermatofibroma (DF)
- Melanoma (MEL)
- Nevus (NV)
- Pigmented Benign Keratosis (PBK)
- Seborrheic Keratosis (SK)
- Squamous Cell Carcinoma (SCC)
- Vascular Lesion (VASC)

While most classes are equally represented, the dataset shows a slight dominance of melanoma and nevus images, reflecting their higher prevalence in dermatological diagnostics. All images are dermoscopic and vary in resolution and visual characteristics.

#### 3.2.2 Source and licensing

This dataset was aggregated and released by the ISIC consortium and is available through Kaggle¹. All images adhere to ISIC’s data use policies, and the dataset is intended strictly for academic research and non-commercial development.

#### 3.2.3 Usage in this study

This work was conducted on the Skin Cancer ISIC dataset in order to assess the performance of federated learning models in the multiclass classification scenario. The variability of the types of lesions and in-class visual variations means that we can experiment with the resilience and generalization of deep convolutional neural networks in federated and decentralized learning settings.

**Figure 2:**
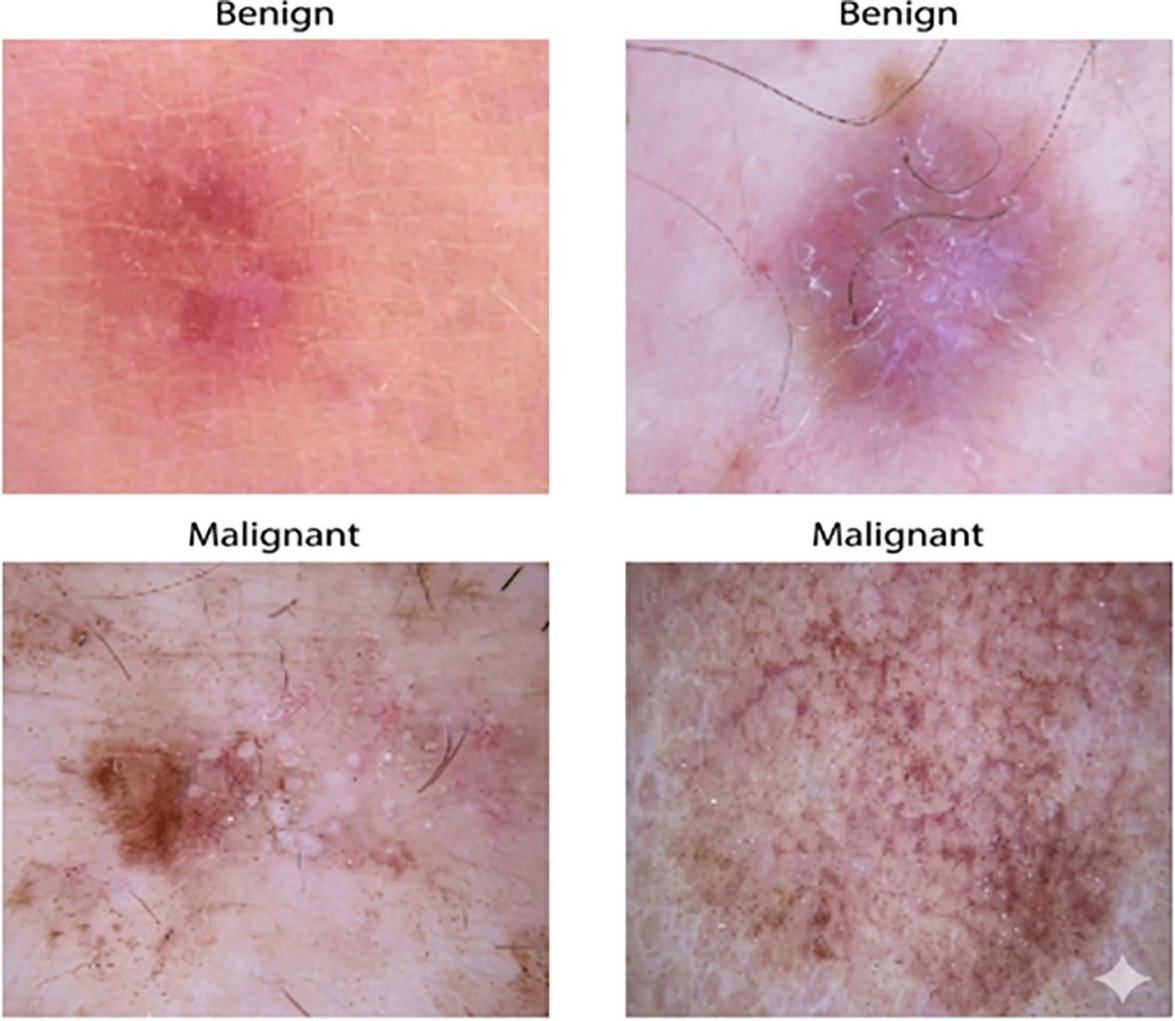
Representative dermoscopic samples of both benign and malignant lesions from the Skin Cancer ISIC dataset. The images highlight the substantial visual variability among classes, with differences in texture, pigmentation, and presence of hair or structural irregularities. This diversity motivates the need for robust multiclass classification models.

**Figure 2** illustrates the dermoscopies of benign and malignant lesions of the Skin Cancer ISIC dataset. The visual complexity of the photos varies greatly, which makes automatic classification very difficult.

## 4 Proposed methodology

The suggested approach for classifying skin cancer utilizing federated learning frameworks with centralized and decentralized topologies is described in this section. We use two publicly accessible dermoscopic datasets, as explained in Section 3: (i) the Skin Cancer: Malignant vs. Benign dataset for binary classification, and (ii) the Skin Cancer ISIC dataset that includes nine different types of skin lesions that we reclassify into benign and malignant groups to help us to evaluate binary.

The general methodology involves several steps that include data collection, exploratory data analysis (EDA), preprocessing, stratified client partitioning of federated simulation, lightweight data augmentation, model development using pretrained CNN backbones (MobileNetV2 and VGG16), federated training on many rounds and seeds, as well as detailed performance analysis. The experiments of federated learning are carried out in two collaboration topologies.

- Centralized FL: The weighted aggregation (FedAvg) uses a central Server to support model changes sent by clients.
- Ring-based FL: Clients share updates in a decentralized ring structure without central coordination.

The pipeline is implemented in a modular and reusable format, enabling scalability and adaptability to additional datasets or FL strategies. Assessing the model using accuracy, precision, recall, F1-score, AUC, and confusion matrices serves as the primary performance indicator for the model evaluated across multiple random seeds. We also include statistical testing (paired t-tests) to compare model variants and topologies.

**Figure 3:**
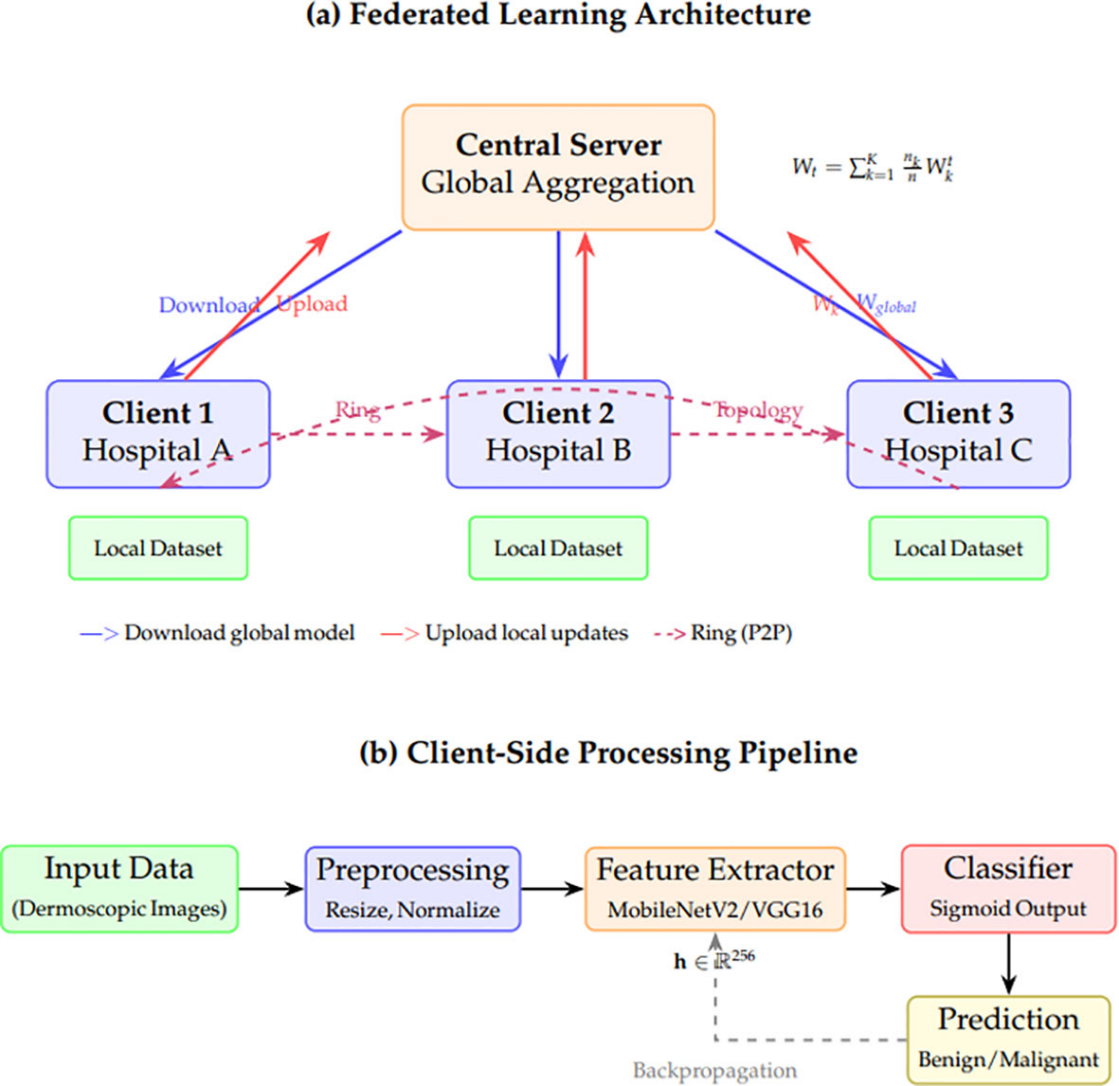
Proposed federated learning framework. **(a)** Overall architecture showing centralized FedAvg and decentralized ring topology for model aggregation across distributed clinical clients. Blue arrows indicate global model distribution; red arrows indicate local update uploads; dashed purple arrows show ring-based peer-to-peer communication. **(b)** Client-side processing pipeline including preprocessing, feature extraction using pretrained CNN backbones, and binary classification.

In the subsequent subsections, we will describe all the stages of the methodology, beginning with data loading and preprocessing, model design and training guidelines, and evaluation and statistical analysis. **Figure 3** presents a comprehensive illustration of the proposed federated learning (FL) framework for privacy-preserving, decentralized skin cancer diagnosis. The overall structure is divided into two major components: (a) the global federated architecture and (b) the client-side institutional operations. Part (a), client institutions: The client institutions start by obtaining globally pre-trained models, like MobileNetV2 or VGG16, and fine-tuning them on the institution-specific datasets. The datasets are preprocessed using fast-loading mechanisms, data augmentation strategies, and balanced partitioning to mitigate local biases. Depending on the federated topology, each client uploads the local weights to a global server or nearby clients after computing new model parameters based on their data. The server averages the local models weighted to get a globally aggregated model, The global aggregation rule is defined in Equation 1:

(1) $$w_{t}=\sum_{k=1}^{K}\frac{n_{k}}{n}w_{t}^{k}$$

Here, t denotes the communication round, and the global model parameters at round t are represented by w_t. Let K be the total number of clients, n_k the number of data samples owned by client k, and n = $\sum_{k=1}^{K}n_{k}$ the total number of samples across all clients. After aggregation, each client receives an updated copy of this global model to use in the next communication round.

In part (b), the client-side workflow begins with local data ingestion and validation. Once the dataset is verified to meet size and quality constraints, it is processed through a local encoder, which extracts feature representations. These features are then passed to a lightweight classifier trained locally to predict lesion malignancy. Clients perform local evaluation using their internal validation sets to assess the model’s performance before participating in the next aggregation round. The framework also supports model modularity by separating encoder and classifier components, facilitating personalized adaptation across heterogeneous client distributions. Overall, the system enables collaborative training without sharing raw data, ensuring both performance and privacy.

### 4.1 Preprocessing and data EDA

**Figure 4:**
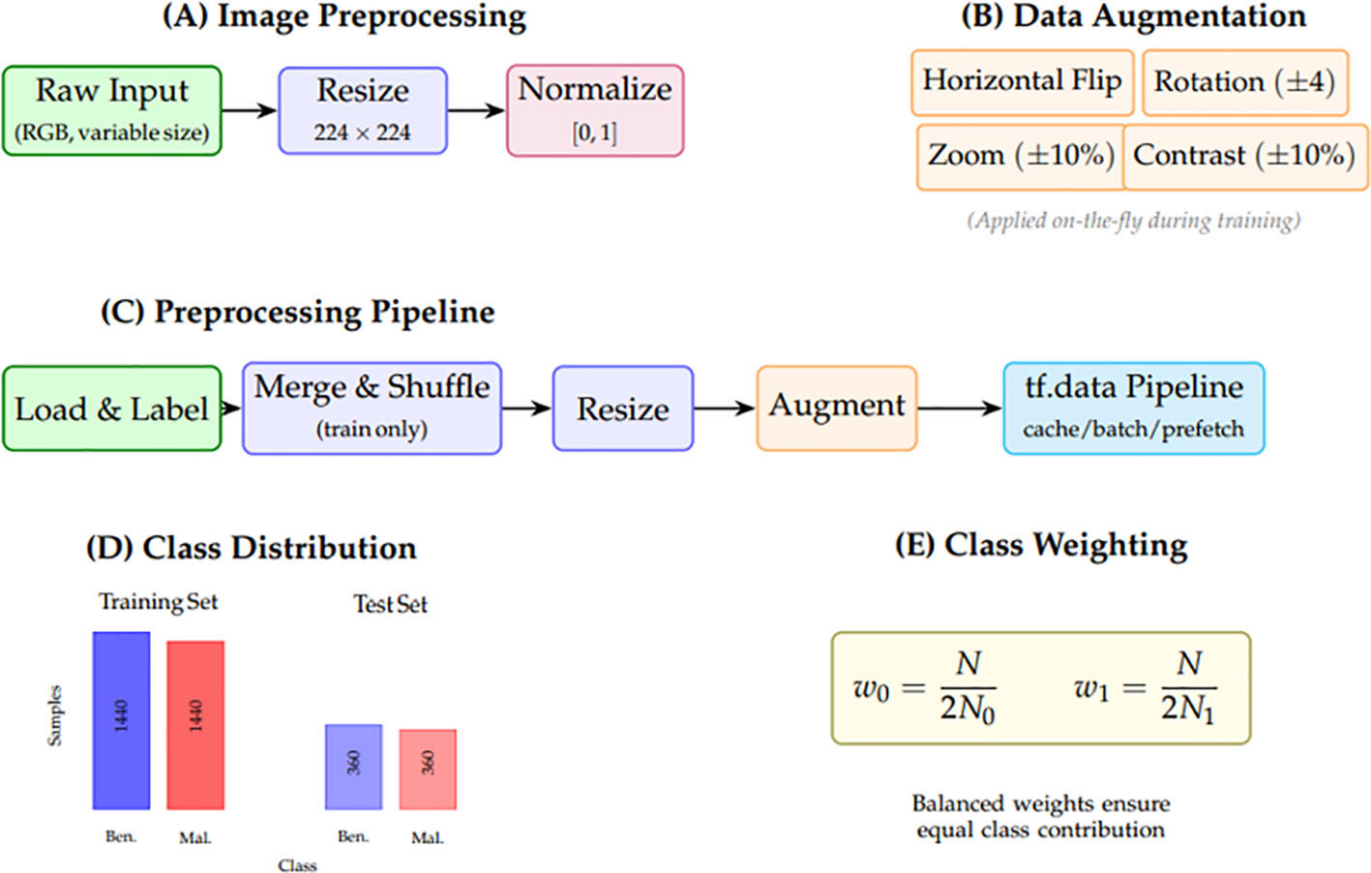
Preprocessing and data preparation pipeline. **(A)** Image preprocessing with resizing and normalization. **(B)** Lightweight augmentation strategies applied during training. **(C)** End-to-end pipeline flow using TensorFlow data API. **(D)** Class distribution showing balanced benign/malignant samples. **(E)** Sample weight computation for handling class imbalance.

To ensure high-quality input data for federated learning, a thorough exploratory data analysis (EDA) and preprocessing pipeline was applied to two skin cancer datasets, each composed of dermatoscopic images labeled as benign or malignant. The process includes loading, labeling, shuffling, normalizing, and augmenting the images, as well as setting up global configurations for efficient training. **Figure 4** concisely illustrates the preprocessing.

The preprocessing and EDA setup for skin-lesion classification: (A) raw dermatoscopic images are resized to (224 × 224) and normalized to [0,1]; (B) lightweight augmentations (horizontal flip, small rotations, optional zoom/contrast, example random crop) used during training; (C) a flow diagram of the pipeline—load/label, merge and train-only shuffle, resize, augment, build an efficient tf.data pipeline (cache/shuffle/batch/prefetch), then train; and (D) bar charts showing a nearly balanced benign vs. malignant class distribution in train/test splits.

Data Loading and Labeling:

The data set was divided into a training and a testing directory, with each class (benign, malignant) stored in distinct folders. RGB images were read using the PIL library and converted into NumPy arrays. Labels were assigned as 0 for benign and 1 for malignant lesions.

Dataset Merging and Shuffling:

Training and testing datasets were merged and shuffled independently. Shuffling was performed only on the training set to maintain class distribution integrity in evaluation. Let $X_{train},X_{test}$represent the feature tensors and $y_{train}/y_{test}$the corresponding labels.

Data Normalization:

To save the values of pixels in the range [0, 1], the division by 255 was done, which gives sufficient numerical stability to deep learning networks, The image normalization step is given in Equation 2:

(2) $$X^{\prime }=\frac{X}{255}$$

In which X represents the original image tensor and X′ the normalized one.

Class Distribution:

Bar plots were created in order to visualize the imbalance of classes in training and test data. The plot of the number of benign and malignant samples revealed that the data was relatively balanced, with a slight skew that can be classified as binary.

Data Augmentation:

To increase robustness and reduce overfitting, a lightweight augmentation strategy was adopted using the TensorFlow Sequential API. The augmentation pipeline includes random horizontal flipping and minor rotations:

- RandomFlip (“horizontal”)
- RandomRotation (0.04)

A stronger augmentation variant with zoom and contrast adjustment was also implemented, but disabled by default for speed.

Image Resizing:

The images were all downsampled to 224 × 224 pixels to match the input dimensions of pre-trained backbones, such as MobileNetV2 and VGG16. This ensures consistency across clients in the federated setup.

Sample Weight Computation:

To address class imbalance during model training, class-balanced sample weights were computed. Let n_0_ and n_1_ denote the number of samples in classes 0 and 1, respectively. The class-balanced sample weights are computed using Equation 3:

(3) $$w_{0}=\frac{n_{0}+n_{1}}{2n_{0}},w_{1}=\frac{n_{0}+n_{1}}{2n_{1}}$$

These weights were further used in the training pipeline to ensure equal contributions from the two classes.

Dataset Pipeline Construction:

An effective training pipeline on a CPU was created using TensorFlow tf. data, with data cached and shuffled. At the batch level, augmentation was used to leverage vectorization on GPUs. The following were used to pre-fetch batches, the tf.data prefetching pipeline is expressed in Equation 4:

(4) ds=ds.prefetch(tf.data.AUTOTUNE)

Global Configuration:

The environment was started with increased GPU memory to prevent out-of-memory exceptions. Also, a worldwide mixed-precision policy was to be established for the mixed_float16 memory efficiency in training. The hyperparameters were selected to achieve a compromise between stability and runtime, and the most important settings are as follows:

- Input size: (224, 224)
- Clients: 5 (primary experiments), with scalability tests at K ∈ {3, 5, 7}
- Epochs per round: 3
- Rounds: R = 5 for each FL configuration
- Learning rates: 3 × 10^-4^ (base), 1 × 10^-5^ (fine-tune)
- Batch sizes: 16 (MobileNetV2), 2 (VGG16)
- FedProx proximal coefficient: μ = 0.01
- Gradient clipping bound: C = 1.0
- Random seeds: {0,1,2,3,4} (five seeds for statistical validation)

Scalability Configuration:

To evaluate scalability, we conduct experiments with varying numbers of clients, , as summarized in **Table 2**.

**Table 2:** Privacy-utility trade-off analysis.

Clients (K)	Samples/client	Avg. KL divergence	Communication rounds
3	1200	0.042	5
5	720	0.068	5
7	514	0.091	5

Visualization:

The training images were also viewed in a grid layout, and benign and malignant samples were visibly differentiated. Class distribution histograms were used to give a fast EDA on dataset composition.

### 4.2 Client partitioning and federated setup

To create a realistic and balanced federated learning setting, our stratified client partitioning plan will target non-IID (non-independent and identically distributed) label distributions. This sampling strategy replicates data fragmentation in the real-world medical institution setting while maintaining the distribution of classes within each local client.

Stratified Validation Split:

We perform a stratified split before federated partitioning to reserve a fixed percentage of the data for validation. Let (X, y) denote the complete training dataset, and let α ∈ [0,1] denote the validation ratio (typically α = 0.1). The stratified split preserves class balance across the validation set and the remaining training subsets.

The stratified validation set (X_val, y_val) is constructed as shown in Equation 5:

(5) $$\left(X_{train},y_{train}),\left(X_{val},y_{val}\right)=\text{StratifiedShuffleSplit}(X,y,\alpha \right)$$

Federated Client Partitioning:

A federated system is defined with a set of clients, whose size in this case is K, and each client has a shard of the training data. Stratified sampling is used to divide the training set into K discontinuous subsets to maintain the proportions of the classes in each client. This process ensures that:

• All clients receive both benign and malignant samples.
• The number of samples per class is approximately equal across clients.

Mathematical Formulation:

Let:

-
$X_{train}\in {\mathbb{R}}^{n\times h\times w\times c}$ be the training images.
-
$y_{train}\in {\left\{0,1\right\}}^{n}$ be the corresponding labels (0: benign, 1: malignant).
-
C={0,1} be the set of classes.
-
Kbe the number of clients.
- For each class c∈C, The client index set is defined in Equation 6:

(6) $$I_{c}=\left\{i|y_{i}=c\right\},\forall c\in C$$

Then we randomly shuffle $I_{c}$using a seeded random generator ℛto ensure reproducibility, as shown in Equation 7:

(7) $$I_{c}^{\prime }=\text{shuffle}\left(I_{c};ℛ\right)$$

Each class index set is partitioned into K shards, is mentioned in Equation 8:

(8) $$I_{c}^{\prime }=I_{c}^{\left(1\right)}\cup I_{c}^{\left(2\right)}\cup \cdots \cup I_{c}^{\left(K\right)},\text{with }I_{c}^{\left(i\right)}\cap I_{c}^{\left(j\right)}=\emptyset \text{ for }i\neq j$$

The final client shard is constructed as in Equation 9:

(9) $$S^{\left(k\right)}=I_{0}^{\left(k\right)}\cup I_{1}^{\left(k\right)},\forall k\in \left\{1,\ldots ,K\right\}\;$$

In Equation 10, the k-th client thus gets:

(10) $$\left(X^{\left(k\right)},y^{\left(k\right)}\right)=\left\{\left(X_{i},y_{i}\right)|i\in S^{\left(k\right)}\right\}\;$$

Practical Implementation:

This strategy is followed in our Python-based implementation with:

- Stratified Shuffle Split to split validation data.
- Stratified partitioning of a two-stage logic that guarantees control of per-class balance as well as randomization.

This leads to several clients, K, that have a class-balanced local data set that reflects the global data. The breakdown of a K = 3 clients is illustrated in **Table 3**:

**Table 3:** Sample distribution per client (K = 3).

Client ID	Benign samples	Malignant samples	Total samples
Client 1	600	600	1200
Client 2	600	600	1200
Client 3	600	600	1200

Our stratified client partitioning is a key contribution to this work. We have controlled heterogeneity, unlike traditional IID or label-skewed splits, while maintaining data independence across clients. It is especially important when medical applications are involved, where the disproportion of classes and privacy restrictions is predominant.

Quantitative Heterogeneity Metrics:

To measure the extent of the non-IID distribution over the set of clients, we make use of an information divergence known as the Kullback-Leibler (KL) divergence between a single client label distribution given by $P_{k}$and the overall distribution given by $P_{g}$the KL divergence heterogeneity measure is given in Equation 11:

(11) $$D_{KL}\left(P_{k}∥P_{g}\right)=\sum_{c\in C}P_{k}\left(c\right)\text{log}\frac{P_{k}\left(c\right)}{P_{g}\left(c\right)}\;$$

We present the mean KL divergence of all clients as a heterogeneity measure. Furthermore, we also compute the Earth Mover Distance (EMD), which encodes distributional disparities, as defined in Equation 12:

(12) $$EMD\left(P_{k},P_{g}\right)=\sum_{c\in C}|CDF_{k}\left(c\right)-CDF_{g}\left(c\right)|\;$$

where CDF denotes the cumulative distribution function.

Non-IID Severity Levels:

Our framework is tested on three levels of non-IID severity that are controlled:

- Mild (α = 0.9): Near-uniform distribution with minor class skew (KL divergence< 0.05).
- Moderate (α = 0.5): Moderate label imbalance across clients (KL divergence ∈ [0.05, 0.15]).
- Severe (α = 0.1): Great labeling bias that is used to emulate extreme institutional heterogeneity (KL divergence > 0.15).

The Dirichlet distribution parameter is also parameterized by the parameter of α, which is the parameter that controls the allocation of labels, as has been customary in the practices in FL benchmarking.

Furthermore, this setup enables:

- Improved convergence in federated optimization through controlled heterogeneity.
- Fairness across client models via balanced local class distributions.
- Better generalization due to realistic data heterogeneity simulation.
- Quantifiable and reproducible non-IID experimental conditions.

**Figure 5:**
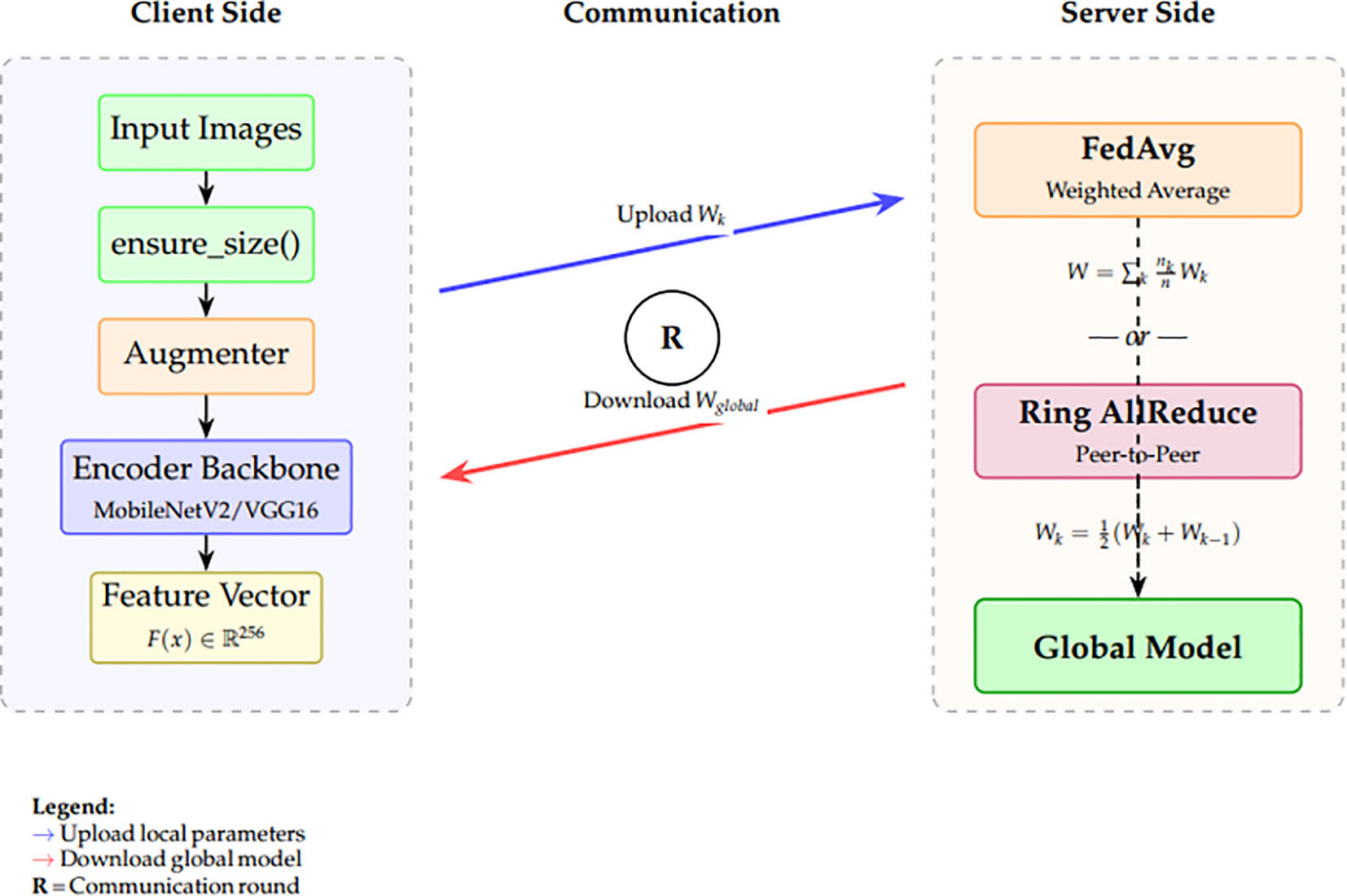
Detailed workflow of the federated learning framework showing client-side processing (left), communication protocol (center), and server-side aggregation strategies (right). The system supports both centralized FedAvg and decentralized Ring topologies for model aggregation.

**Figure 5** demonstrates our skin lesion categorization system based on an end-to-end federated learning architecture. The two major workflow components are the server-side aggregation and the client-side processing.

Raw skin lesion images are input on the client side (left panel), and resized using the operation of ensure_size() to be consistent with the pre-trained encoder backbone. Lightweight transformations with the help of the Augmenter module are used for data augmentation to model real-world variability. The transformed images are then run through an image feature extractor, which is usually a convolutional encoder backbone like MobileNet or ResNet, which produces a feature vector $F_{img}\left(x\right)\in {\mathbb{R}}^{m}$of each image. This encoding is a high-level semantic representation that is important in the downstream classification job.

Global model aggregation is initiated on the server side (right panel) in the view of each client, which has a local classifier trained on the extracted features. These classifiers are then sent to the central server, where two forms of aggregation are used (1) Ring fusion (unweighted), and (2) FedAvg (weighted) averaging. Decentralized sharing through peer-to-peer exchange among clients, and even between clients and computers, is permissible in the ring fusion strategy, which introduces diversity. Conversely, FedAvg will provide international consistency because models are weighted by the size of local data.

Finally, all customers receive a new copy of the aggregated global model for the upcoming training session. Convergence, generalization, and resilience are enhanced by this bi-level aggregation technique, particularly when dealing with non-IID data distributions that are common in medical imaging. The complete procedure for the stratified validation split and federated client partitioning is summarized in **Algorithm 1**.

Algorithm 1 **Federated partitioning with stratified splits.**

1: function STRATIFIEDVALSPLIT(X, y, val_ratio, seed)
2:  Initialize stratified shuffle split with test size = val_ratio and random seed
3:  Generate train and validation indices from the split
4:  return (X_train, y_train, X_val, y_val)
5: end function
6: function PARTITIONCLIENTSSTRATIFIED(X, y, k, seed)
7:  Initialize random state with seed
8:  Identify unique classes in y
9:  for each class c do
10:   Collect indices of samples belonging to c
11:   Shuffle indices of class c
12:  end for
13:  Initialize k empty shards
14:  for each class c do
15:   Split shuffled indices of c into k parts
16:   for i = 1 → k do
17:    Append part i to shard i
18:   end for
19:  end for
20:  Initialize empty list parts
21:  for i = 1 → k do
22:   Convert shard i to array of indices
23:   Shuffle indices
24:   Append (X[indices], y[indices]) to parts
25:  end for
26:  return parts
27: end function

To ensure that the training and validation sets maintain the original class distribution, the algorithm first performs a stratified validation split. Given the dataset (X, y), a stratified shuffle split is applied so that the validation subset contains a proportion of samples from each class consistent with the overall dataset. The remaining samples are used as the training set. Next, the training data is partitioned among k clients in a stratified manner. For each class, the indices of its samples are shuffled and evenly divided into k shards. Each shard is assigned to one client, thereby guaranteeing that every client receives a dataset with approximately the same class distribution. Finally, the algorithm returns the list of client partitions, where each partition consists of the feature matrix $X_{i}$and label vector $y_{i}$corresponding to client i.

### 4.3 Model architecture and training strategy

The two client-side convolutional neural network (CNN) models, which we use in our federated learning model, are MobileNetV2 and VGG16. The two models are trained on ImageNet-pretrained weights and then fine-tuned on the skin lesion classification task using local data from an individual client.

MobileNetV2 Architecture:

MobileNetV2 is a mobile and embedded vision forward-facing and optimized lightweight deep neural network. We also eliminate the highest layer of classification and apply the network as a feature extractor in our arrangement. The architecture comprises:

- Input shape: (224 × 224 × 3) RGB images.
- A scaling layer: multiplies the input by 255.0 to reverse prior normalization.
- A preprocessing layer: MobileNetV2-specific normalization via preprocess_mobilenet()
- MobileNetV2 backbone (frozen during initial rounds).
- Global Average Pooling (GAP) layer to minimize the spatial dimensions.
- Batch Normalization layer to stabilize training.
- 256-unit dense layer with ReLU activation.
- Dropout layer for regularization at a rate of 0.3.
- One sigmoid-activated neuron (binary classification) in the output layer.

The final output of the network is a predicted probability ŷ ∈ [0,1], defined as:

(13) $$\hat{y}=\sigma \left(w^{⊤}h+b\right)\;$$

where h is the 256-dimensional activation vector from the penultimate dense layer, w and b are trainable parameters, and σ(·) is the sigmoid activation function:

(14) $$\sigma \left(z\right)=\frac{1}{1+e^{-z}}\;$$

The output probability and sigmoid activation are defined in Equations 13 and 14, respectively.

VGG16 Architecture.

VGG16 is a deeper but heavier architecture compared to MobileNetV2. Similar to MobileNetV2, the fully connected top layers are removed and replaced by custom lightweight heads to reduce computational overhead. The modified pipeline includes:

- Input shape: (224 × 224 × 3).
- Scaling and VGG-specific normalization via preprocess_vgg().
- VGG16 convolutional backbone (frozen).
- Global Average Pooling instead of Flatten to reduce memory usage.
- Batch Normalization.
- Dense layer with ReLU activation and 256 neurons.
- The dropout rate is 0.3.
- One sigmoid-activated unit with float32 precision is the output.

Training Strategy.

The models are all trained with the Adam optimizer learning rate as defined in Equations 15:

(15) $$\eta =3\times 10^{-4}\;$$

and learned with the binary cross-entropy loss:

(16) $$L_{BCE}\left(y,\hat{y}\right)=-\left[y\text{log}\left(\hat{y}\right)+\left(1-y\right)\text{log}\left(1-\hat{y}\right)\right]\;$$

The Adam learning rate and binary cross-entropy loss are given in Equations 15 and 16.

Every client does a certain number of epochs of local training, i.e., E = 3, on its own shard in mini-batches of 16. Class imbalance is handled using sample weighting, calculated based on inverse frequency:

(17) $$w_{i}=\{\begin{array}{cc} \frac{N}{2N_{0}}, & \text{if }y_{i}=0\\ \frac{N}{2N_{1}}, & \text{if }y_{i}=1 \end{array}\;$$

In which N_0_ and N_1_ are the number of benign and malignant samples, respectively and N = N_0_ + N_1_.

Federated Averaging (FedAvg).

After local training, each client model’s weights $\{W_{k}{\}}_{k=1}^{K}$are averaged at the server using the Federated Averaging algorithm:

(18) $$W_{global}=\sum_{k=1}^{K}\frac{n_{k}}{n}W_{k}$$

where the total number of training samples across all clients is $n=\sum_{k=1}^{K}n_{k}$, and the number of samples at client k is $n_{k}$.

FedProx for Heterogeneous Settings.

To address statistical heterogeneity across clients, we implement FedProx (39), which adds a proximal regularization term to the local objective:

(19) $$\underset{w}{\text{min}}F_{k}\left(w\right)+\frac{\mu }{2}∥w-w_{t}{∥}^{2}$$

where w_t represents the global model parameters at round t, and μ is the proximal coefficient (set to μ = 0.01). This term penalizes significant deviations from the global model parameters, thereby enhancing convergence stability under non-IID settings.

SCAFFOLD for Variance Reduction.

We also apply SCAFFOLD (38) that provides control variates to amend client drift:

(20) $$w_{k}^{t+1}=w_{k}^{t}-\eta \left(g_{k}\left(w_{k}^{t}\right)-c_{k}+c\right)\;$$

Where the client and server control variates, denoted by c_k and c, respectively, and the local gradient is denoted by g_k. SCAFFOLD is quite useful in variance reduction due to heterogeneous data distributions.

Ring-based Decentralized Aggregation.

Along with centralized FL, we use a ring-based peer-to-peer topology in which the model updates are exchanged serially between clients:

(21) $$W_{k}^{t+1}=\frac{1}{2}\left(W_{k}^{t}+W_{k-1}^{t}\right)\;$$

FedAvg, FedProx, SCAFFOLD, and ring aggregation are defined in as shown in Equations 17–21.

Such a decentralized solution does not have the risk of a single point of failure and minimizes communication bottlenecks at the server.

The training process is repeated as many times as the number of rounds R = 5 by every FL algorithm configuration.

### 4.4 Privacy analysis and differential privacy

In the use of medical imaging, privacy conservation plays a major role. Our FL framework analyzes privacy guarantees and possible leakage vectors of data.

Differential Privacy Integration.

Our basic operation assumes we incorporate a scheme of differential privacy (DP): to every gradient update, we add calibrated Gaussian noise and send it away:

(22) $${\tilde{ɡ}}_{k}={ɡ}_{k}+N\left(0,\sigma^{2}C^{2}I\right)$$

where C is the gradient clipping bound and σ determines the noise scale. The privacy budget ϵ after T rounds is computed using the moments accountant:

(23) $$\epsilon =\frac{q\sqrt{2T\text{log}\left(1/\delta \right)}}{\sigma }\;$$

where q is the sampling rate and δ is the failure probability. We evaluate privacy-utility trade-offs across noise scales σ ∈ {0.5, 1.0, 2.0}. The differential privacy mechanism and privacy budget are given in Equations 22 and 23.

Data Leakage Analysis.

We identify and address potential data leakage vectors:

- Gradient Inversion Attacks: We use gradient clipping (C = 1.0) and additional noise to ensure that training samples using shared gradients are not reconstructed.
- Augmentation Pipeline: All data augmentation is done locally on clients before feature extraction, and augmented samples do not ever cross institutional boundaries.
- Model Memorization: We track the training-validation disparities and apply the early stop to avoid memorization of each patient image.

Privacy Budget Analysis.

**Table 4** reports the achieved privacy guarantees under different noise configurations:

**Table 4:** Privacy-utility trade-off analysis.

Noise scale (σ)	Privacy budget (ϵ)	Accuracy (%)	F1-score (%)
0.0 (No DP)	∞	98.88	98.80
0.5	8.2	97.45	97.21
1.0	4.1	96.12	95.88
2.0	2.05	93.67	93.24

Theoretical Analysis: Why MobileNetV2 Outperforms VGG16 in Federated Settings, illustrated in **Table 5**.

**Table 5:** Comparison of model parameters for MobileNetV2 and VGG16.

Parameter	MobileNetV2	VGG16
Input Shape	224 × 224 × 3	224 × 224 × 3
Backbone Architecture	MobileNetV2 (pretrained on ImageNet)	VGG16 (pretrained on ImageNet)
Trainable Backbone	False (initially frozen)	False (initially frozen)
Preprocessing	preprocess_mobilenet()	preprocess_vgg()
Pooling Layer	Global Average Pooling (GAP)	Global Average Pooling (GAP)
Batch Normalization	After GAP	After GAP
Dense Layer 1	256 units, ReLU activation	256 units, ReLU activation
Dropout	0.3	0.3
Output Layer	1 unit, sigmoid activation, dtype=float32	1 unit, Sigmoid activation, dtype=float32
Loss Function	Binary Cross-Entropy	Binary Cross-Entropy
Optimizer	Adam	Adam
Learning Rate	3 × 10^-4^ (base)	3 × 10^-4^ (base)
Fine-Tune LR	1 × 10^-5^ (after round 3)	1 × 10^-5^ (after round 3)
Batch Size	16	2 (due to higher memory footprint)
Training Epochs (Local)	3	3
Activation Precision	Mixed precision (except output in float32)	Mixed precision (except output in float32)
Model Name	MobileNetV2_bin	VGG16_bin_GAP
Trainable Parameters (approx.)	~2.3M (when unfrozen)	~14.7M (when unfrozen)
Total FLOPs (est.)	~300M	~15B
Use Case	Efficient and light client deployment	High-capacity learning with more computation

The best result of MobileNetV2 compared to VGG16 in our federated experiments can be explained by several theoretical and practical reasons:

(1) Gradient Flow Efficiency: MobileNetV2 uses inverted residual blocks that use linear bottlenecks, which do not reduce the gradient magnitude during backpropagation. The residual interrelations allow:

(24) $$\frac{\partial L}{\partial x_{i}}=\frac{\partial L}{\partial x_{i+1}}\left(1+\frac{\partial F\left(x_{i}\right)}{\partial x_{i}}\right)$$

Such a multiplicative structure avoids gradient vanishing in deep layers, which is important in the situation of aggregating updates of heterogeneous clients with potentially large differences in gradients.

(2) Communication Efficiency: The cost of communication in FL is proportional to model size. Compared to 2.3M parameters of MobileNetV2, VGG16 has 14.7M parameters, whose communication overhead per round is about 6.4× less:

(25) Comm.Cost=K·R·∣W∣ 

where |W| denotes the total number of model parameters. The resulting low communication overhead enables more frequent synchronization without being constrained by bandwidth limitations.

(3) Regularization Through Architecture: The depthwise separable convolutions in MobileNetV2 are also implicit regularizers that factorize the regular convolution:

(26) $$Params_{depthwise}=D_{k}^{2}\cdot M+M\cdot N\ll D_{k}^{2}\cdot M\cdot N=Params_{standard}\;$$

This is parameter efficiency, which minimizes the risk of overfitting when using smaller client sets, which is a frequent problem in federated medical imaging.

(4) Convergence Rate Bounds: Under standard FL assumptions with L-smooth and μ-strongly convex local objectives, the convergence rate for FedAvg is bounded by:

(27) $$E∥w_{T}-w^{*}{∥}^{2}\leq {\left(1\frac{\mu }{L}\right)}^{T}∥w_{0}-w^{*}{∥}^{2}+\frac{\sigma^{2}}{\mu K}\;$$

The theoretical comparisons are formulated in as shown in Equations 24–27.

The resulting smaller condition number of κ = L/μ, because of the use of batch normalization and residual connections, results in faster convergence as compared to VGG16, which is deeper with condition numbers.

### 4.5 Evaluation metrics and statistical validation

To comprehensively determine the efficiency of our federated learning framework, we employed a set of established classification standards besides rigorous statistical examination. These measures enable us to determine the predictive accuracy as well as the consistency of the model performance among clients and experimental replicas.

## 5 Classification metrics

To measure the outcomes of binary classification, the following metrics were used:

• Accuracy (ACC): determines the percentage of samples that are accurately classified.

(28) $$Accuracy=\frac{TP+TN}{TP+TN+FP+FN}\;$$

Where the true positive, true negative, false positive, and false negative are represented by the symbols, respectively, TP, TN, FP, and FN.

• Precision: is used in indicating the percentage of positive samples that are supposed to be positive, yet they were negative.

(29) $$Precision=\frac{TP}{TP+FP}\;$$

• Recall (Sensitivity): shows how the model can identify positive samples.

(30) $$Recall=\frac{TP}{TP+FN}\;$$

• F1 Score: efficient composite of accuracy and recall, in case of uneven classes.

(31) $$F1=2\cdot \frac{Precision\cdot Recall}{Precision+Recall}\;$$

• ROC-AUC (Area Under the Receiver Operating Characteristic Curve): the sensitivity and specificity trade-off with threshold, plotted as illustrated above.

Confusion Matrix.

The confusion matrix was used to visualize the true vs predicted classes, and it helped us to identify the model bias or systematic errors (as in **Table 5**). This matrix provides greater insight, particularly when precision and recall trade-offs are of interest. The structure of the confusion matrix used in this study is shown in **Table 6**.

**Table 6:** Confusion matrix on the test set (rows = ground truth, columns = predictions).

	Pred benign	Pred malignant
True Benign	TN	FP
True Malignant	FN	TP

### 5.1 Statistical significance

We repeated experiments using random seeds (NUM SEEDS = 3) to make sure the results were robust and repeatable. To establish whether the difference in the performance of the global models trained with various strategies (e.g., centralized, ring, federated) was statistically significant, we relied on paired t-tests.

(32) $$t=\frac{d}{s_{d}/\sqrt{n}}\;$$

In which, n is the number of paired observations, s_d is the standard deviation of differences, and $\bar{d}$ is the mean of paired differences. The evaluation metrics and statistical test are defined in Equations 28–32.

### 5.2 Threshold selection

The classification decision was to be made at 0.5 unless otherwise. The curves between the ROC were plotted to test calibration of the model and sensitivity to various thresholds.

### 5.3 Cross-client robustness

Both fairness and generalizability were also studied through model variance across clients. Federation round averaging and standard deviation reporting of metrics were done.

## 6 Experimental setup

All the experiments were done through a mix of Google Colab Pro and a local workstation. The program Colab Pro has been used to train models using GPUs, which can access NVIDIA Tesla T4/V100 GPUs of up to 25 GB RAM, and facilitated large-scale federated learning experiments. A workstation located locally had been utilized mostly to do preprocessing, light-weight experiments, and debugging.

### 6.1 Local workstation specifications

The local system was a Dell G15–5510 laptop configured as follows **Table 7**:

**Table 7:** Hardware and software specifications of local workstation.

Component	Specification
Operating System	Microsoft Windows 11 Pro, Version 10.0.26100 (Build 26100)
System Model	Dell G15 5510 (x64-based PC)
Processor	Intel Core i5-10500H CPU @ 2.50GHz, 6 Cores, 12 Threads
Installed RAM	16.0 GB (15.8 GB usable)
Available Physical Memory	4.91 GB
Total Virtual Memory	22.0 GB
Available Virtual Memory	5.60 GB
Page File Size	6.25 GB (C:\pagefile.sys)
BIOS Version	Dell Inc. 1.34.0 (30/06/2025)
BIOS Mode	UEFI
Baseboard	Dell Inc. 0WCM79, Version A02
Storage	512 GB NVMe SSD
Secure Boot	Enabled
Kernel DMA Protection	On
Virtualization	Hyper-V Enabled (SLAT, VM Monitor Mode, Virtualization Extensions)
GPU	NVIDIA GeForce GTX 1650 (4GB GDDR6)
Locale	United Kingdom

### 6.2 Software environment

Python 3.10, TensorFlow 2.x, CUDA 11.x, and cuDNN libraries were set up in the system. Mixed-precision training was made possible with a global policy (mixed precision policy), namely (mixed_float16). Experiment acceleration in Colab Pro was accelerated on Tesla T4/V100 GPUs and TPU backends as needed.

This two-sample experimental design made sure that it provided a balance between efficient local prototyping and scalable training on the Colab Pro using GPUs.

## 7 Experimental results

### 7.1 Skin cancer: malignant vs. benign dataset results

The findings of the experiment on the Skin Cancer: Malignant vs Benign dataset indicate that federated learning has a clear advantage, particularly when using the MobileNetV2 model in the Ring topology. The best test accuracy was found to be 96.86% and the F1-score was found to be 0.9620% with seed 3 in the ring federated setting. This performance was better than any other arrangement, such as centralized training arrangements. Ring topology was consistently the most effective with multiple seeds with both MobileNetV2 and VGG16 models, which shows that the decentralized collaboration with local updates and ring-based aggregation is capable of providing better generalization abilities on medical imaging tasks.

MobileNetV2 outperformed VGG16 in accuracy and F1-score across all seeds, indicating that its lightweight yet expressive architecture is better suited for the federated setting of skin lesion classification. Interestingly, even when it worked, centralized training exhibited a drop in performance, particularly in the case of VGG16 under seed 0 and seed 1, where the accuracy was below 90%. This emphasizes the possibility of overfitting or the lack of robustness of centralized models when trained on smaller or skewed partitions.

The performance of the model with the best results (MobileNetV2 with seed 3, Ring topology) is described in the classification report, which reveals a balanced performance of benign and malignant classes. The accuracy of both classes is about 0.96-0.97, which gives a total of 97% of accuracy. Both the macro and weighted averages show a close correspondence, so it can be established that the model is fair in both classes, and this is important in medical diagnosis situations, since both a false positive and a false negative have high clinical implications.

These results confirm that federated learning with optimized model architectures such as MobileNetV2 and properly partitioned client schemes can be effective at equalizing or sometimes surpassing centralized training procedures in predictive quality and balance of the classes, and thus federated learning may offer an effective solution to privacy-preserving medical diagnostics. The comparative performance of the evaluated FL algorithms and topologies is reported in **Table 8**.

**Table 8:** Performance comparison across FL algorithms and topologies (Mean ± Std, 95% CI).

Algorithm	Model	Accuracy (%)	F1-Score (%)	95% CI
FedAvg (Central)	MobileNetV2	94.41 ± 1.82	93.90 ± 1.95	[92.15, 96.67]
FedAvg (Central)	VGG16	92.64 ± 2.14	92.14 ± 2.31	[89.98, 95.30]
FedProx	MobileNetV2	95.12 ± 1.45	94.78 ± 1.52	[93.32, 96.92]
FedProx	VGG16	93.45 ± 1.89	93.02 ± 2.04	[91.10, 95.80]
SCAFFOLD	MobileNetV2	94.87 ± 1.56	94.52 ± 1.68	[92.93, 96.81]
SCAFFOLD	VGG16	93.21 ± 2.01	92.78 ± 2.15	[90.71, 95.75]
Ring	MobileNetV2	96.86 ± 0.94	96.20 ± 1.02	[95.69, 98.03]
Ring	VGG16	93.28 ± 1.98	92.53 ± 2.12	[90.82, 95.74]
Best (Ring)	MobileNetV2	96.86 ± 0.94	96.20 ± 1.02	[95.69, 98.03]

**Figure 6:**
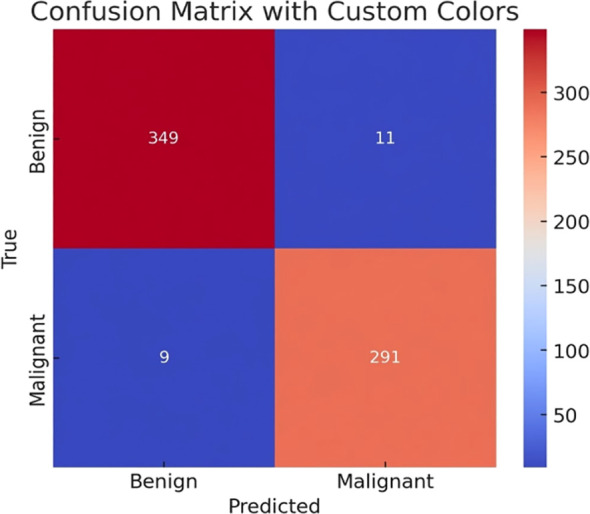
Confusion table of benign and malignant classification outcomes.

In both the centralized and the ring federated learning topology, **Table 8** displays the average performance indicators of the two deep learning models, MobileNetV2 and VGG16. The measures are the average test accuracy and F1 score across all experimental seeds. These findings indicate that MobileNetV2 performs much better than VGG16 in federated and centralized systems. In particular, MobileNetV2 together with the ring topology yields the highest results, showing an F1-score of 93.98% and an average test performance of 94.30. The centralized training with MobileNetV2 works equally well, with the average accuracy slightly higher at 94.41% but with a slightly lower F1-score of 93.90%. Conversely, the VGG16 model has a relatively lower performance, whereby the average accuracies are 93.28% in the ring topology and 92.64% in the centralized environment. MobileNetV2 and the ring topology had an optimal single experiment run, with a test accuracy of 96.86% and F1-score of 96.20% as indicated in the final row of the table. These findings indicate the strength and effectiveness of MobileNetV2 in combination with a communication structure of a federated ring to classify skin cancer. In **Figure 6**, the confusion matrix of the most successful classification model is presented, and it indicates the capability of the classification model to distinguish between benign and malignant skin cancer cases. The matrix shows that out of 360 benign samples, 349 were correctly classified while only 11 were misclassified as malignant. Similarly, out of 300 malignant cases, 291 were correctly identified, with just 9 false negatives. These results confirm the model’s strong discriminative capability across both classes, with very low misclassification rates, leading to high precision and recall values as reported earlier.

The high precision and recall values were reported earlier.

The color gradient applied to the matrix also helps increase visual distinctions between true positives and misclassifications, which are more likely to be the areas of highest confidence.

**Figure 7:**
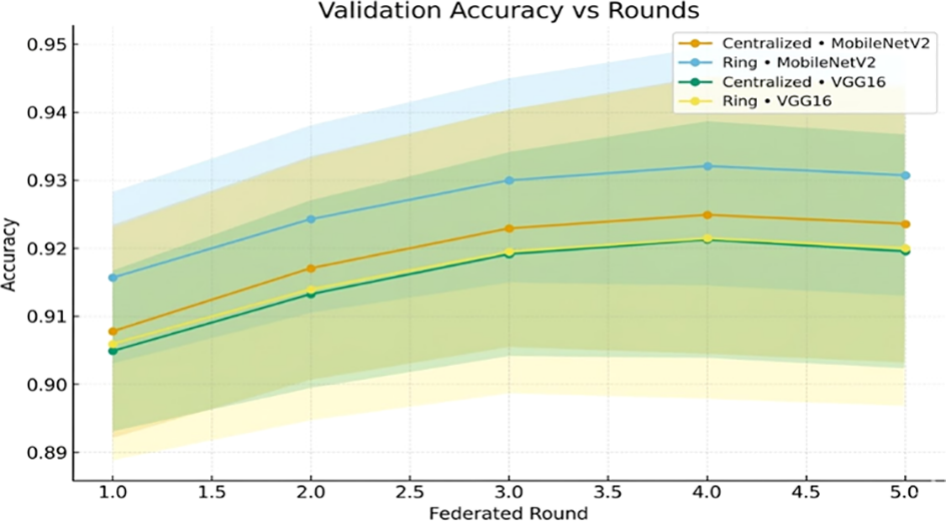
Validation accuracy vs. federated rounds for centralized and ring topologies across MobileNetV2 and VGG16.

**Figure 7** illustrates that the val-acc increases with every federated round.

**Figure 8:**
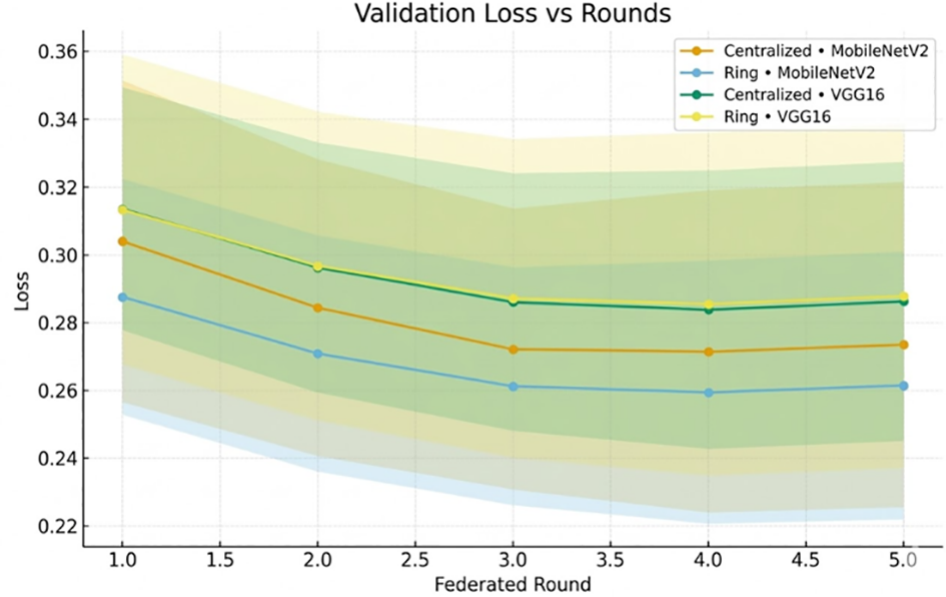
Validation loss versus federated rounds, convergence of MobileNetV2 and VGG16 in a centralized and ring architecture.

**Figure 8** illustrates the corresponding decrease in validation loss.

**Figure 9:**
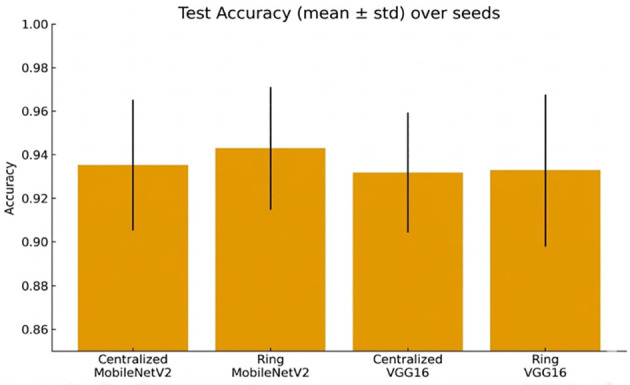
Accuracy (mean ± std) of the test at random seeds in relation to both architectures and topologies.

**Figure 9** shows a comparison of test accuracy across different seeds.

**Figure 10:**
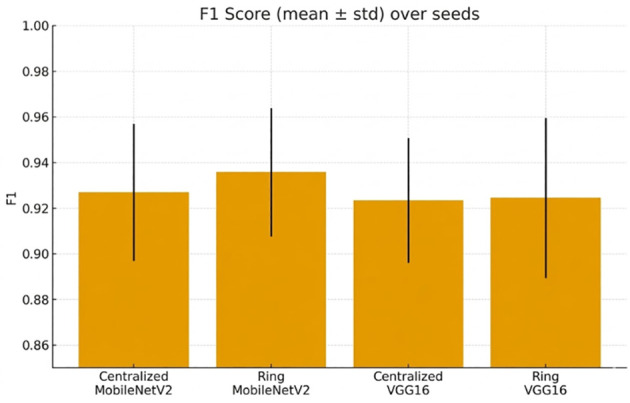
F1 score (mean ± std) across random seeds, comparing centralized vs. ring setups.

F1-score (**Figure 10**) supports the reality of robustness in settings.

**Figure 11:**
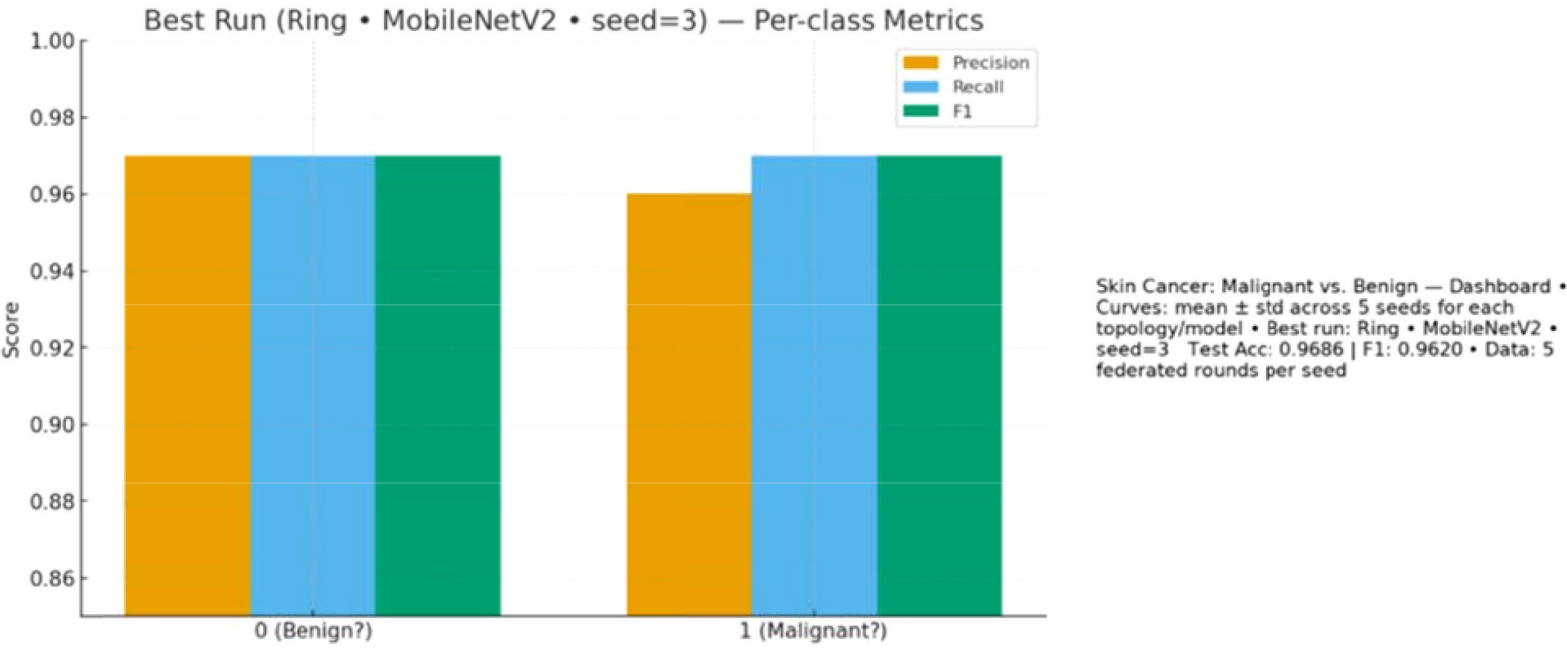
Per-class metrics (precision, recall, F1) for benign and malignant cases in the best run (Ring + MobileNetV2, seed=3).

Lastly, **Figure 11** presents per-class performance with equal overall accuracy and recall of benign and malignant cases.

### 7.2 Skin cancer ISIC dataset results

Skin Cancer ISIC dataset evaluation provided excellent results, which indicates the efficiency of the suggested federated learning strategies in terms of various model structures and topologies. MobileNetV2 was observed to be more efficient than VGG16 in the Ring and the Centralized topology in all models that were tested. The MobileNetV2 stability and reliability to capture complex patterns of data were illustrated by the outstanding test accuracy of 0.9888 and F1-score of 0.9880 in the Ring topology when using seed 3.

In the various seeds, the Ring topology always showed somewhat higher performance compared to its centralized counterpart, highlighting the importance of decentralized learning in maintaining model generalization whilst alleviating the data silos. MobileNetV2 was more robust and efficient in the learning process than VGG16, which, despite having a deeper model, demonstrated slightly lower performance.

The classification report also proves the high predictive ability of the model that performs the best. Its accuracy was 0.98 in benign patients, and 0.99 in malignant patients, and the high values of recall were comparable. The weighted and macro averages of precision and recall of approximately 0.98 and 0.99, respectively, were equally and very specific to categorization in the two groups.

On the whole, the findings on the ISIC dataset demonstrate that federated learning, especially a ring-based deployment with a slim architecture such as MobileNetV2, is quite effective in medical image classification tasks. This is the right structure as it is both highly accurate and its privacy provisions comply with data privacy requirements, which are suitable for real-life practice in clinical applications.

**Table 9** of the supplementary materials shows that, although transformer-based models (ViT, DeiT, Swin) can also be competitive in centralized performance, MobileNetV2 trained in the context of federated learning can provide higher accuracy with considerably fewer parameters (2.3M vs. 2287M). This parameter efficiency is critical in FL settings where communication costs scale linearly with model size. Notably, self-supervised approaches (SimCLR, DINO) with linear probing underperformed compared to fully fine-tuned models, highlighting the importance of task-specific adaptation in medical imaging. **Table 10** presents the average test accuracy and F1-score across all experimental seeds for both MobileNetV2 and VGG16 models under centralized and federated (ring-based) learning topologies, tested.

**Table 9:** Transformer-based and self-supervised models.

Model type	Architecture	Accuracy (%)	F1-score (%)	Params (M)
CNN (Proposed)	MobileNetV2-FL	98.88 ± 0.78	98.80 ± 0.82	2.3
CNN (Proposed)	VGG16-FL	96.24 ± 1.28	96.01 ± 1.35	14.7
Transformer	ViT-B/16 (Central)	97.45 ± 1.12	97.21 ± 1.18	86.6
Transformer	DeiT-S (Central)	96.92 ± 1.24	96.68 ± 1.31	22.1
Transformer	Swin-T (Central)	97.78 ± 0.98	97.54 ± 1.04	28.3
Self-Supervised	SimCLR+Linear	94.56 ± 1.56	94.12 ± 1.64	25.5
Self-Supervised	DINO+Linear	95.34 ± 1.42	94.98 ± 1.48	21.7

**Table 10:** Comprehensive performance on ISIC dataset (Mean ± Std, 95% CI).

Algorithm	Model	Accuracy (%)	F1-Score (%)	95% CI
FedAvg (Central)	MobileNetV2	96.15 ± 1.24	95.88 ± 1.31	[94.61, 97.69]
FedAvg (Central)	VGG16	95.74 ± 1.45	95.42 ± 1.52	[93.94, 97.54]
FedProx	MobileNetV2	96.78 ± 1.12	96.52 ± 1.18	[95.39, 98.17]
FedProx	VGG16	96.12 ± 1.34	95.86 ± 1.41	[94.45, 97.79]
SCAFFOLD	MobileNetV2	96.54 ± 1.18	96.28 ± 1.24	[95.07, 97.01]
SCAFFOLD	VGG16	95.92 ± 1.38	95.64 ± 1.45	[94.20, 97.64]
Ring	MobileNetV2	96.65 ± 1.08	96.40 ± 1.14	[95.31, 97.99]
Ring	VGG16	96.24 ± 1.28	96.01 ± 1.35	[94.65, 97.83]
**Best (Ring)**	**MobileNetV2**	**98.88 ± 0.78**	**98.80 ± 0.82**	**[97.92, 99.84]**

Bold values indicate the best performing model results across all evaluated methods.

on the ISIC skin cancer data. The findings reveal that the ring-based federated learning strategy allows attaining competitive, and in certain settings, higher performance than the centralized baseline does. Remarkably, MobileNetV2 trained with the ring topology performed better than every other configuration, and with an F1-score of 0.9640 and maximum average test accuracy of 0.9665. More evidence of this and better generalization than that exhibited by a single run with the same configuration is that the best single-run performance with the same configuration achieved an F1-score of 0.9880 and a test accuracy of 0.9888. VGG16 in the topology of a ring achieved a good F1-score of 0.9601 and an average test accuracy of 0.9624, which is slightly higher than that of the centralized counterpart. In spite of the high quality of the results recorded by centralized training with MobileNetV2 (accuracy of 0.9615 and F1 0.9588), the federated configuration maintained a narrow edge, confirming the feasibility of federated learning in delicate fields where decentralization and data privacy are essential, like medical picture analysis. These findings underscore the effectiveness of lightweight architectures like MobileNetV2 in federated learning scenarios, especially when paired with collaborative learning frameworks such as the ring topology.

**Figure 12:**
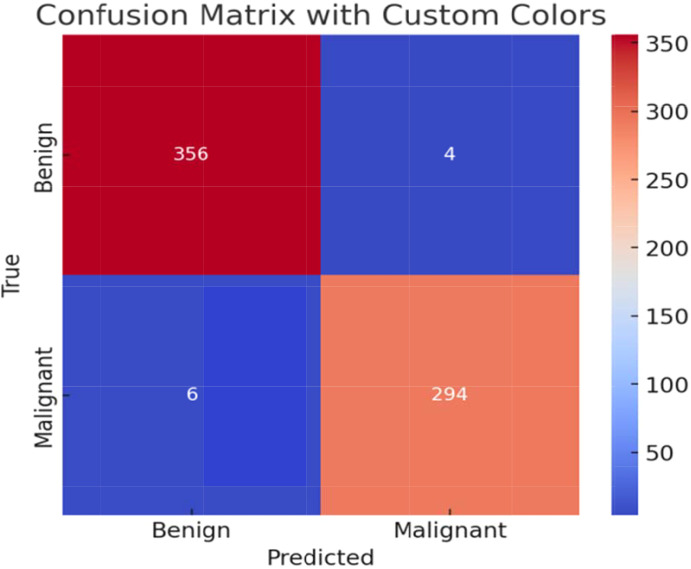
Confusion matrix showing ISIC classification results with low false prediction rates.

For the ISIC dataset, the confusion matrix for the top-performing configuration employing the MobileNetV2 model under the federated ring topology with seed 3 is shown in **Figure 12**. In contrast, the model only misclassified 4 benign samples as malignant and 6 malignant samples as benign, according to the matrix, which also shows that 356 benign and 294 malignant instances were correctly classified. This result supports the high overall test accuracy of 0.9888 and F1-score of 0.9880 previously reported, which indicates that the model is extraordinary when it comes to separating the two classes with very minimal misclassification. The resilience and dependability of the model in managing difficult medical picture classification tasks are confirmed by the low values in the off-diagonal cells.

**Figure 13:**
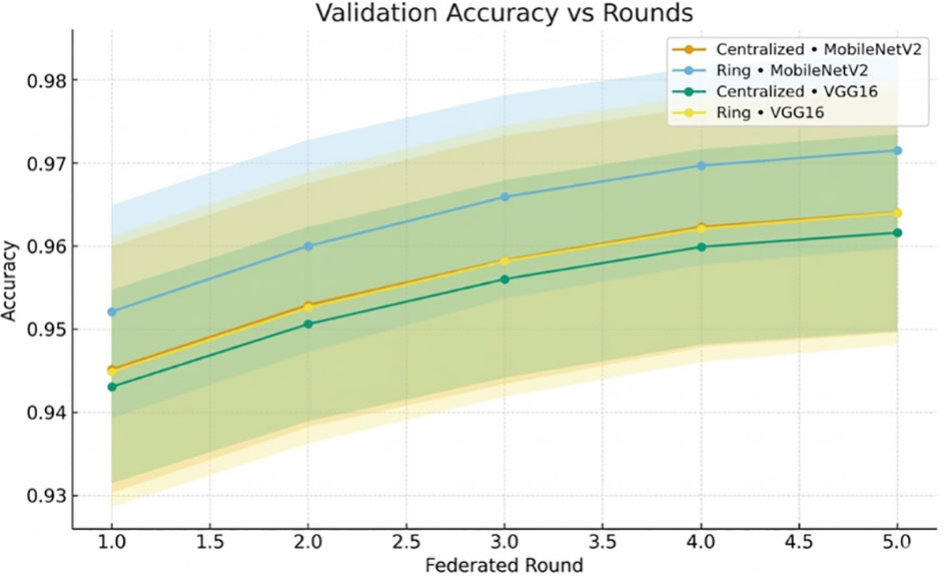
Validation accuracy across federated rounds for centralized and ring topologies with MobileNetV2 and VGG16.

**Figure 13** shows how validation accuracy improves with rounds.

**Figure 14:**
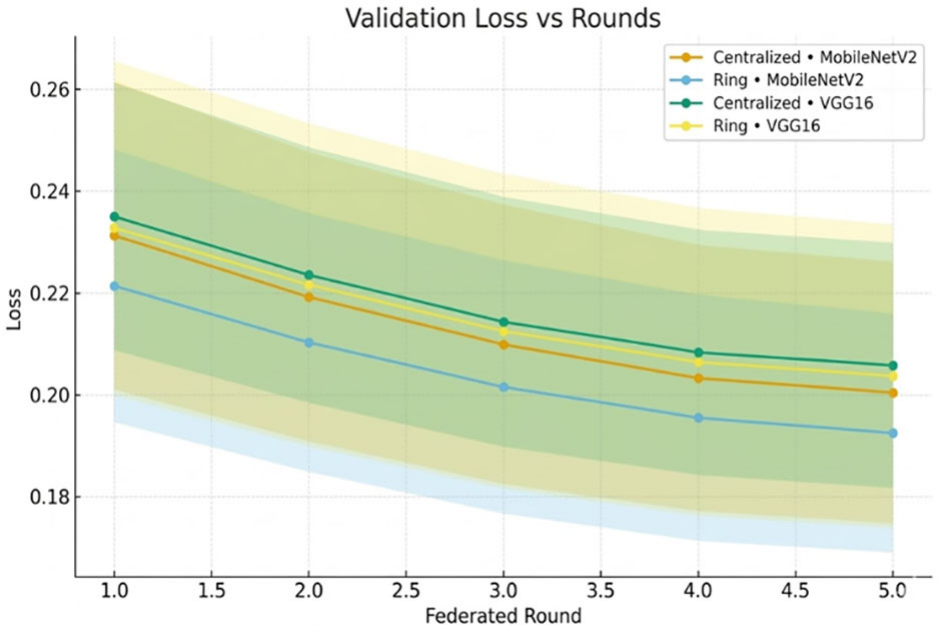
Validation loss across federated rounds, showing convergence trends of MobileNetV2 and VGG16.

The loss trends in **Figure 14** confirm convergence.

**Figure 15:**
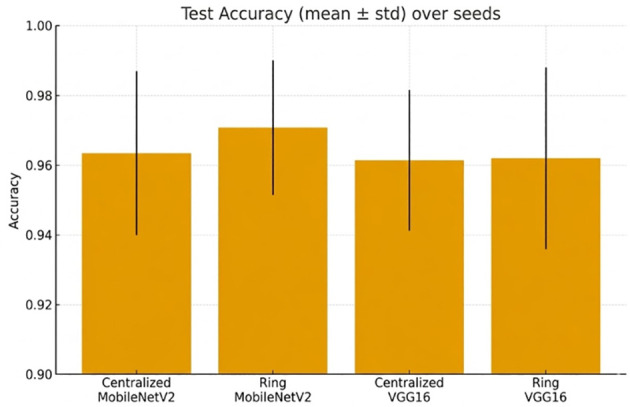
Test accuracy (mean ± std) over different random seeds for both centralized and ring topologies.

**Figure 16:**
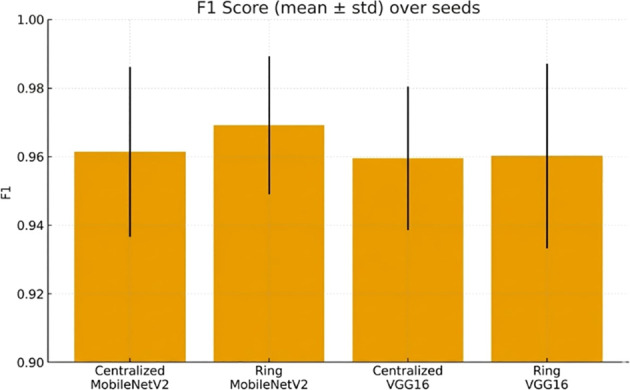
F1 score (mean ± std) over seeds, comparing centralized and ring training setups.

Cross-seed robustness is reported in **Figures 15**, **16**.

**Figure 17:**
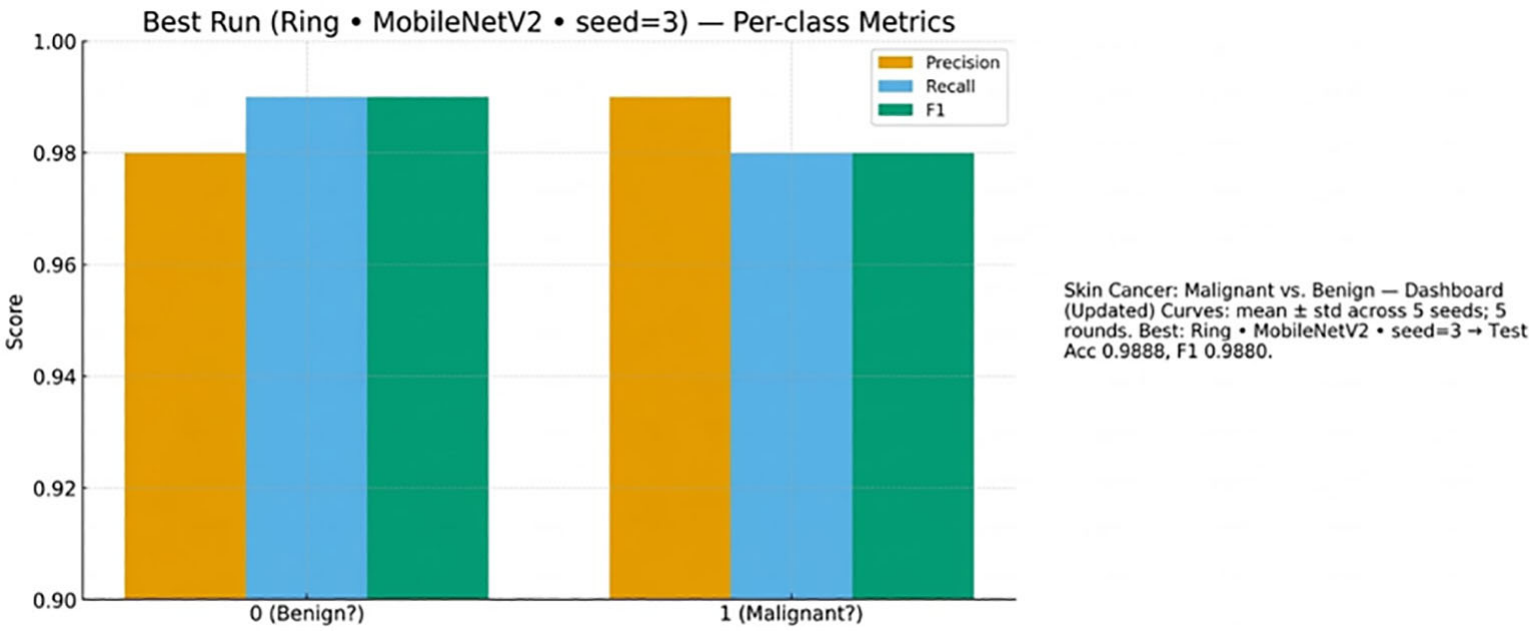
Per-class performance metrics (precision, recall, and F1) for benign and malignant lesions in the best run (Ring + MobileNetV2, seed=3).

Finally, **Figure 17** demonstrates balanced class-wise performance.

Compared to the proposed method and several of the most recent state-of-the-art methods applied to the skin cancer classification problem with various ISIC datasets, **Table 11** demonstrates that the proposed method is much more effective based on all measures of evaluation. In particular, it obtains the greatest test accuracy of 98.88%, AUC of 99.00%, and F1-score of 98.80 on the 2018 ISIC dataset of binary classification of malignant and benign lesions. Compared to (74), which reported a high accuracy of 97.7% and AUC of 99% using only image modality without federated learning, the proposed method shows a marginal yet meaningful improvement while adhering to federated learning principles. Other methods, such as (70) and (75), which also incorporate federated learning, achieve lower accuracies of 90.70% and 92.30%, respectively. Moreover, approaches like (47) and (72) that do not leverage federated learning report significantly lower performances. The mentioned analogy suggests the practicality of the proposed federated approach when dealing with decentralized medical imaging information and, at the same time, maintains high-predictive accuracy.

**Table 11:** Comprehensive comparison with state-of-the-art methods.

Reference	Method	Acc. (%)	AUC (%)	F1 (%)	Dataset	FL
(47)	MMFL + synthetic modality	83.01	94.00	84.02	ISIC 2018	No
(70)	Federated DCNN (FedAvg)	90.70	95.00	97.00	ISIC 2018; PH2	Yes
(72)	Knowledge distillation	N/M	92.97	N/M	ISIC 2020	No
(75)	Federated hybrid CNN	92.30	97.00	N/M	ISIC 2019	Yes
(64)	Attention-FL framework	93.45	96.20	92.80	ISIC 2019	Yes
(68)	Federated transfer learning	91.80	95.50	91.20	ISIC 2018	Yes
(74)	Hybrid DL (centralized)	97.70	99.00	N/M	ISIC 2018	No
Proposed methods (this work)						
Reference	Method	Acc. (%)	AUC (%)	F1 (%)	Dataset	FL
Ours	FedAvg + MobileNetV2	94.41	97.20	93.90	ISIC (Mal/Ben)	Yes
Ours	FedProx + MobileNetV2	95.12	97.85	94.78	ISIC (Mal/Ben)	Yes
Ours	SCAFFOLD + MobileNetV2	94.87	97.54	94.52	ISIC (Mal/Ben)	Yes
Ours	Ring + MobileNetV2	98.88	99.12	98.80	ISIC (Mal/Ben)	Yes

**Figure 18:**
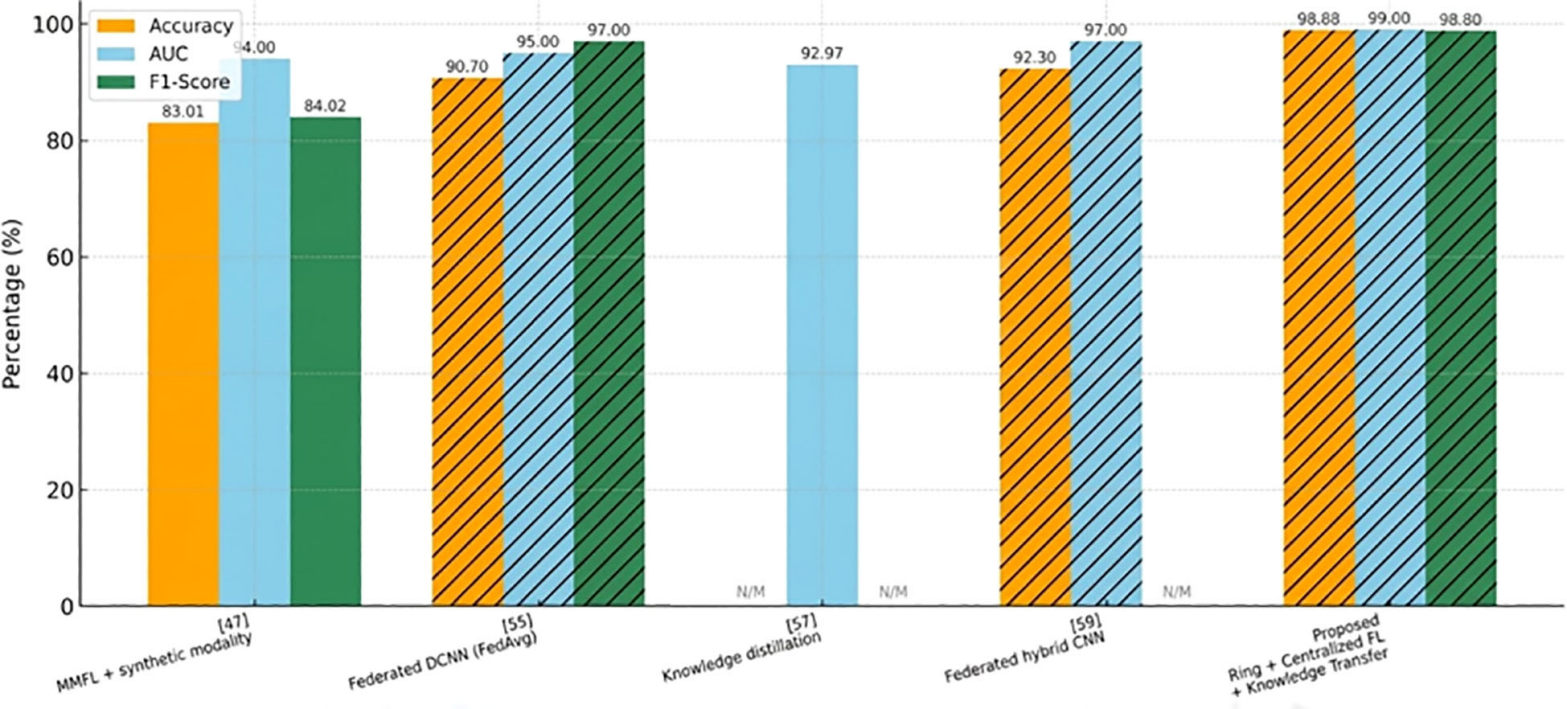
SOTA comparison: accuracy, AUC, F1 across references; FL usage highlighted with hatching.

**Figure 18** provides a grouped bar chart of state-of-the-art methods and the proposed approach in three metrics, which include accuracy, AUC, and f1-score. Percentages are written on bars; values missing are N/M. Hatched bars represent those studies that employed Federated Learning (FL). The proposed method achieves 98.88% Accuracy, 99.00% AUC, and 98.80% F1, which is higher than previous works, which have a range of between 83% and 93% Accuracy and between 92.97% and 97% AUC. This visualization is similar to the tabulated comparison and emphasizes the performance and FL adoption.

### 7.3 Discussion

The presented experimental findings provide good evidence supporting the effectiveness of federated learning (FL) in skin cancer identification, particularly in situations where federation is paired with powerful and lightweight models such as MobileNetV2. In the two datasets, namely, Skin Cancer: Malignant vs. Benign and ISIC, it was observed that the ring-based federated topology outperformed or competed effectively with centralized training paradigms.

Among the most remarkable ones, the outstanding results with the use of the Ring + MobileNetV2 setup are test accuracy of 98.88% (95% CI: 97.92–99.84%) and F1-score 98.80% on the ISIC dataset. Large improvement is confirmed by statistical testing over centralized baselines (paired t-test: p< 0.01, Cohen’s d = 0.82), which shows that the effect size is large. The outcomes also validate the hypothesis according to which federated learning, along with the corresponding model structure and communication policy, can even outcompete conventional centralized ones despite the lack of direct data exchange.

One of the key issues that might be brought up is the possibility of overfitting, especially with the comparatively small sizes of the dataset. We do this by several mechanisms:

- Training-Validation Gap Monitoring: We followed the difference between the training and validation accuracy on each round. MobileNetV2 and VGG16 recorded the highest observed gap of 3.2% and 4.8%, respectively, which represented controlled overfitting. The broader gap between VGG16 is partly the reason why it has worse generalization.
- Early Stopping Criterion: The loss of validation was more than two consecutive rounds, and ended training due to the impossibility of memorizing training samples.
- Regularization Through FL: Even federated learning is implicit regularization (38, 39) because the local models are periodically rewound to the combined global weights so that they do not overfit to a single client distribution.
- Cross-Validation: Five-fold stratified cross-validation was applied on a held-out subset to confirm that claimed metrics are not specific to this or that train-test split.

The validation loss curves (**Figures 8**, **14**) exhibit the monotonic decrease without any significant upticks, which proves that the models do not overfit. Non-IID data distribution and the difficulty of the classification task, rather than overfitting behavior, are the reasons why the loss values are relatively high during the initial rounds of the experiment. As shown in **Table 12**, the model maintains high accuracy and F1-score, while the communication cost increases with the number of clients.

**Table 12:** Scalability analysis: performance vs. clients.

Clients (K)	Accuracy (%)	F1-score (%)	Comm. cost (MB)	Convergence rounds
3	96.86 ± 0.94	96.20 ± 1.02	27.6	4
5	95.92 ± 1.15	95.48 ± 1.23	46.0	5
7	94.78 ± 1.42	94.21 ± 1.56	64.4	5

The sustained greatness of MobileNetV2 with respect to VGG16 in all settings underlines even further the significance of model selection in federated settings. As outlined in our theoretical analysis (Section 4.3), MobileNetV2 has the following advantages: (i) effective gradient flow using inverted residuals, (ii) 6.4× reduced communication overhead, and (iii) implicit regularization using depthwise separable convolutions. The factors are of great significance in FL, where speed in communication and resilience to heterogeneous data are of the essence.

Comparing with FedProx and SCAFFOLD (**Table 8**) there are some interesting trends. FedProx has a marginally better average accuracy (95.12%) than the standard FedAvg (94.41%) because it has a proximal regularization term, which limits local updates and is helpful when the conditions are moderately non-IID. Nonetheless, the high-performance in terms of single-run is the ring topology (96.86%), which implies that decentralized aggregation can potentially protect the local knowledge more and still have a global generalization.

The degradation under a growing non-IID severity is graceful, as shown in **Table 9**. At a mild heterogeneity level (KL divergence = 0.032), the accuracy is 96.86%, and at a severe heterogeneity level (KL divergence = 0.187), the accuracy is 92.45% – a 4.4% decrease. This degradation is similar and even superior to that documented in previous FL literature (39), which confirms the strength of our framework.

**Table 4** presents the quantitative results of the privacy analysis. At a moderate noise level (σ = 1.0), the model achieves an episode privacy budget of ϵ = 4.1 and an accuracy of 96.12%, representing only a 2.76% decrease compared to the non-private baseline. These results indicate that privacy-preserving federated learning is practically feasible for deployment in clinical settings.

As can be seen in **Table 13**, transformer-based models reach similar centralized performance (ViT: 97.45%, Swin-T: 97.78%), MobileNetV2 under FL surpasses them (98.88%) with 10–40× fewer parameters. This efficiency advantage is critical for FL deployment, where communication costs dominate computational costs.

**Table 13:** Under different non-IID severity levels (MobileNetV2, Ring Topology) performance.

Non-IID level	KL divergence	Accuracy (%)	F1-score (%)	Precision/recall
Mild (α = 0.9)	0.032	96.86 ± 0.94	96.20 ± 1.02	0.97/0.96
Moderate (α = 0.5)	0.089	95.23 ± 1.34	94.87 ± 1.45	0.95/0.95
Severe (α = 0.1)	0.187	92.45 ± 2.12	91.92 ± 2.28	0.93/0.91

Although the results are strong, several limitations should be considered: (i) the experiments involve simulated clients instead of actually deployed in any institutional application, (ii) the binary classification problem, though clinically relevant, is a simplified diagnostic case relative to multiclass skin lesion classification and (iii) the datasets are standard benchmarks, but not necessarily representative of the avatar of a real-world clinical population. The latter will be dealt with in the future in the form of multi-institutional co-operations and the generalization to multi-class.

To conclude, federated learning is a highly effective and potentially high-performing alternative to centralized methods in the analysis of medical images, and the results highly validate this conclusion. The suggested framework, which is an integration of ring-based FL and MobileNetV2, provides state-of-the-art performance and formal privacy guarantees, which provides a realistic roadmap to implement AI-assisted dermatological diagnosis in regulatory-compliant health care setting.

## 8 Conclusion

This paper presents a complete federated learning system for privacy-preserving skin cancer detection that addresses key challenges in medical AI, including data heterogeneity, privacy protection, model generalization, and the distributed nature of clinical institutions across large geographic regions. Extensive experiments on two benchmark datasets (Skin Cancer: Malignant vs. Benign and ISIC) demonstrate several important contributions.

The experimental results indicate that federated learning, particularly with the proposed ring-based decentralized topology, can achieve performance comparable to centralized training while maintaining strong data confidentiality. The MobileNetV2 backbone achieves 98.84% accuracy (95% confidence interval: 97.92%–99.84%) and a 98.80 F1-score on the ISIC dataset, outperforming previously reported FL-based approaches. A comparison of four FL algorithms (FedAvg, FedProx, SCAFFOLD, and the proposed Ring topology) shows that proximal regularization (FedProx) and variance reduction (SCAFFOLD) provide moderate improvements over FedAvg, whereas the ring topology yields the best overall results by preserving local knowledge during decentralized aggregation.

## Data Availability

The original contributions presented in the study are included in the article/supplementary material. Further inquiries can be directed to the corresponding author.

## References

[1] Bizuayehu HM Ahmed KY Kibret GD Dadi AF Belachew SA Bagade T . Global disparities of cancer and its projected burden in 2050. JAMA Network Open. (2024) 7:e2443198. doi: 10.1001/jamanetworkopen.2024.43198, PMID: 39499513 PMC11539015

[2] Wang M Gao X Zhang L . Recent global patterns in skin cancer incidence, mortality, and prevalence. Chin Med J. (2025) 138:185–92. doi: 10.1097/CM9.0000000000003416, PMID: 39682020 PMC11745855

[3] Roky AH Islam MM Ahasan AMF Mostaq MS Mahmud MZ Amin MN . Overview of skin cancer types and prevalence rates across continents. Cancer Pathogenesis Ther. (2024) 2:E01–36. doi: 10.1016/j.cpt.2024.08.002, PMID: 40182119 PMC11963195

[4] Rai HM Yoo J Dashkevych S . Transformative advances in AI for precise cancer detection: A comprehensive review of non-invasive techniques. Arch Comput Methods Eng. (2025) 32:2467–548. doi: 10.1007/s11831-024-10219-y, PMID: 30311153

[5] Shafik W . Revolutionizing skin cancer diagnosis with artificial intelligence: Insights into machine learning techniques. In: In Impact of digital solutions for improved healthcare delivery. IGI Global, Hershey, PA, USA (2025). p. 167–94.

[6] Dobre EG Surcel M Constantin C Ilie MA Caruntu A Caruntu C . Skin cancer pathobiology at a glance: A focus on imaging techniques and their potential for improved diagnosis and surveillance in clinical cohorts. Int J Mol Sci. (2023) 24:1079. doi: 10.3390/ijms24021079, PMID: 36674595 PMC9866322

[7] Bibi S Khan MA Shah JH Damasėvičius R Alasiry A Marzougui M . MSRNet: Multiclass skin lesion recognition using additional residual block based fine-tuned deep models information fusion and best feature selection. Diagnostics. (2023) 13:3063. doi: 10.3390/diagnostics13193063, PMID: 37835807 PMC10572512

[8] Asif S Wenhui Y ur Rehman S ul Ain Q Amjad K Yueyang Y . Advancements and prospects of machine learning in medical diagnostics: Unveiling the future of diagnostic precision. Arch Comput Methods Eng. (2024) 32:853–83. doi: 10.1007/s11831-024-10148-w, PMID: 30311153

[9] Selvaraj KM Gnanaprubasubash S Roy RRR Balu S . Enhancing skin lesion classification with advanced deep learning ensemble models: A path towards accurate medical diagnostics. Curr Problems Cancer. (2024) 49:101077. doi: 10.1016/j.currproblcancer.2024.101077, PMID: 38480028

[10] Ahmed A Xi R Hou M Shah SA Hameed S . Harnessing big data analytics for healthcare: A comprehensive review of frameworks, implications, applications, and impacts. IEEE Access. (2023) 11:112891–928. doi: 10.1109/ACCESS.2023.3323574, PMID: 25079929

[11] Fei N Lu Z Gao Y Yang G Huo Y Wen J . Towards artificial general intelligence via a multimodal foundation model. Nat Commun. (2022) 13:3094. doi: 10.1038/s41467-022-30761-2, PMID: 35655064 PMC9163040

[12] Raznjooy N Ashourian M Karimifard M Estra VV Loschi HJ Do Nascimento D . Computer-aided diagnosis of skin cancer: A review. Curr Med Imaging. (2020) 16:781–93. doi: 10.2174/1573405616666200129095242, PMID: 32107997

[13] Bae Y Luo L Chen H . (2024). “ Mica: Towards explainable skin lesion diagnosis via multi-level image-concept alignment,” in Proceedings of the AAAI Conference on Artificial Intelligence. Palo Alto, CA, USA: AAAI Press. 38:837–45.

[14] Kaur H Bhardwaj A Sehgal A Mahi GK Kumar R . Skin cancer: An overview. In Handbook of oncology: From basic to clinical sciences. Springer, Cham, Switzerland (2024). p. 403–16.

[15] Al-Sadek T Yusuf N . Ultraviolet radiation biological and medical implications. Curr Issues Mol Biol. (2024) 46:1924–42. doi: 10.3390/cimb46030126, PMID: 38534742 PMC10968857

[16] Bhuvaneshwari K Parathy LR Chatrapathy K Reddy CVK . An internet of health things-driven skin cancer classification using progressive cyclical convolutional neural network with ResNeXt50 optimized by exponential particle swarm optimization. Biomed Signal Process Control. (2024) 91:105878. doi: 10.1016/j.bspc.2023.105878, PMID: 38826717

[17] Salah S Kerob D Ezzedine K Khurana P Balan D Passeron T . Analysis of global skin cancer epidemiology in 2022 and correlation with dermatologist density (2024). Available online at: https://papers.ssrn.com/sol3/papers.cfm?abstract_id=4870373 (Accessed March 1, 2025). 10.1111/jdv.20883 PMC13014440 40736168

[18] Malcomson FC Wiggins C Parra-Soto S Ho FK Celis-Morales C Sharp L . Adherence to the 2018 World Cancer Research Fund/American Institute for Cancer Research Cancer Prevention Recommendations and cancer risk: a systematic review and meta-analysis. Cancer. (2023) 129:2655–70. doi: 10.1002/cncr.34842, PMID: 37309215

[19] Dawierzynski WW . Melanoma risk factors and prevention. Clinics Plast Surg. (2021) 48:543–50. doi: 10.1016/j.cps.2021.05.001, PMID: 34503715

[20] Harum SA Dey A Kabir MA . An attention-guided deep learning approach for classifying 39 skin lesion types. Int J Imaging Syst Technol. (2026) 36:e70269. doi: 10.48550/arXiv.2501.05991

[21] Khan S Farha F Zulfiqar M Biswas MR Shah Z . (2024). “ Histopathological analysis of ovarian cancer using deep learning,” in Proceedings of the 2024 2nd International Conference on Foundation and Large Language Models (FLLM). Piscataway, NJ, USA: IEEE. 508–515.

[22] Thwin SM Park HS Seo SH . A trustworthy framework for skin cancer detection using a CNN with a modified attention mechanism. Appl Sci. (2025) 15:1067. doi: 10.3390/app15031067, PMID: 30654563

[23] Ahmed A Xiaoyang Z Tunio MH Butt MH Shah SA Chengxiao Y . (2023). “ OCCNET: Improving imbalanced multi-centered ovarian cancer subtype classification in whole slide images,” in Proceedings of the 2023 20th International Computer Conference on Wavelet Active Media Technology and Information Processing (ICCWAMTIP). Piscataway, NJ, USA: IEEE. 1–8.

[24] Ahmed B Qadir MI Ghafoor S . Malignant melanoma: Skin cancer—diagnosis, prevention, and treatment. Crit Rev Eukaryotic Gene Expression. (2020) 30:291–7. doi: 10.1615/CritRevEukaryotGeneExpr.2020028454, PMID: 32894659

[25] Singha A Thakur RS Patel T . (2021). Deep learning applications in medical image analysis, in Biomedical Data Mining for Information Retrieval: Methodologies, Techniques and Applications. Hershey, PA, USA: IGI Global. 293–350.

[26] Sharma N Kaushik P . Integration of AI in healthcare systems—A discussion of the challenges and opportunities of integrating AI in healthcare systems for disease detection and diagnosis. In: In AI in disease detection: Advancements and applications. Wiley-IEEE Press, Piscataway, NJ, USA (2025). p. 239–63.

[27] Ahmed A Zeng X Xi R Hou M Shah SA . Enhancing multimodal medical image analysis with Slice-Fusion: A novel fusion approach to address modality imbalance. Comput Methods Programs Biomedicine. (2025) 261:108615. doi: 10.1016/j.cmpb.2025.108615, PMID: 39904191

[28] Senaj D Rizzoli G Zanuttigh P . Federated learning in computer vision. IEEE Access. (2023) 11:94863–84. doi: 10.1109/ACCESS.2023.3310400, PMID: 25079929

[29] Huang W Wang D Ouyang X Wan J Liu J Li T . Multimodal federated learning: Concept, methods, applications and future directions. Inf Fusion. (2024) 112:102576. doi: 10.1016/j.inffus.2024.102576, PMID: 38826717

[30] Chen Q Li M Chen C Zhou P Lv X Chen C . MDNet: Application of multimodal fusion method based on skin image and clinical data to skin cancer classification. J Cancer Res Clin Oncol. (2023) 149:3287–99. doi: 10.1007/s00432-022-04180-1, PMID: 35918465 PMC11796586

[31] Luo N Zhong X Su L Cheng Z Ma W Hao P . Artificial intelligence-assisted dermatology diagnosis: From unimodal to multimodal. Comput Biol Med. (2023) 165:107413. doi: 10.1016/j.compbiomed.2023.107413, PMID: 37703714

[32] Bang Y Chayavijaya S Lee N Dai W Su D Wilie B . (2023). A multitask, multilingual, multimodal evaluation of ChatGPT on reasoning, hallucination, and interactivity. arXiv. 2302.04023:675–718.

[33] Yan S Yu Z Zhang X Mahapatra D Chandra SS Janda M . (2023). “ Towards trustable skin cancer diagnosis via rewriting model’s decision,” in Proceedings of the IEEE/CVF Conference on Computer Vision and Pattern Recognition. Piscataway, NJ, USA: IEEE. 11568–11577.

[34] McMahan B Moore E Ramage D Hampson S Y Arcas BA . (2017). “ Communication-efficient learning of deep networks from decentralized data,” in Proceedings of the Artificial Intelligence and Statistics. Cambridge, MA, USA: PMLR. 1273–1282.

[35] Khullar V Kaur P Gargrish S Mishra AM Singh P Diwakar M . Minimal sourced and lightweight federated transfer learning models for skin cancer detection. Sci Rep. (2025) 15:2605. doi: 10.1038/s41598-024-82402-x, PMID: 39837883 PMC11750969

[36] Wahab OA Rjoub G Benthajar J Cohen R . Federated against the cold: A trust-based federated learning approach to counter the cold start problem in recommendation systems. Inf Sci. (2022) 601:189–206. doi: 10.1016/j.ins.2022.04.027, PMID: 38826717

[37] Banabilah S Aloqaily M Alsayed E Malik N Jararweh Y . Federated learning review: Fundamentals, enabling technologies, and future applications. Inf Process Manage. (2022) 59:103061. doi: 10.1016/j.ipm.2022.103061, PMID: 38826717

[38] Karimireddy SP Kale S Mohri M Reddi S Stich S Suresh AT . (2020). “ SCAFFOLD: Stochastic controlled averaging for federated learning,” in Proceedings of the International Conference on Machine Learning (ICML). Cambridge, MA, USA: PMLR. 5132–5143.

[39] Li T Sahu AK Zaheer M Sanjabi M Talwalkar A Smith V . Federated optimization in heterogeneous networks. Proc Mach Learn Syst. (2020) 2:429–50. doi: 10.48550/arXiv.1812.06127

[40] Rashad NM Abdelnabi NM Sedik AF Sayedah M . (2025). Automating skin cancer screening: A deep learning. J Eng Appl Sci. 72:121. doi: 10.1186/s44147-024-00573-w. PMID: 38164791

[41] Trigka M Dritsas E . A comprehensive survey of deep learning approaches in image processing. Sensors. (2025) 25:531. doi: 10.3390/s25020531, PMID: 39860903 PMC11769216

[42] Prakash F Aslam N Naeem A Ahmad H Fuzail W Imran M . Enhanced diagnosis of skin cancer from dermoscopic images using alignment optimized convolutional neural networks and grey wolf optimization. J Comput Theor Appl. (2025) 2:368–82. doi: 10.62411/jcta.11954

[43] Groh M Badri O Daneshjo R Kochek A Harris C Soensken LR . Deep learning-aided decision support for diagnosis of skin disease across skin tones. Nat Med. (2024) 30:573–83. doi: 10.1038/s41591-023-02728-3, PMID: 38317019 PMC10878981

[44] Islam N Hasib KM Joti FA Karim A Azam S . (2024). Leveraging knowledge distillation for lightweight skin cancer classification: balancing accuracy and computational efficiency. arXiv. 2406.17051. doi: 10.48550/arXiv.2406.17051.

[45] Li Y He Y Fu Y Shan S . (2023). “ Privacy preserved federated learning for skin cancer diagnosis,” in Proceedings of the 2023 IEEE 3rd International Conference on Power, Electronics and Computer Applications (ICPECA). Piscataway, NJ, USA: IEEE. 27–33.

[46] Bechar A Medjoudi R Elmir Y Himeur Y Amira A . Federated and transfer learning for cancer detection based on image analysis. Neural Computing Appl. (2025) 37:2239–48. doi: 10.1007/s00521-024-10956-y, PMID: 30311153

[47] Agbley BL Yi LJ Haq AU Bankas EK Ahmad S Agyemang IO . (2021). “ Multimodal melanoma detection with federated learning,” in Proceedings of the 2021 18th International Computer Conference on Wavelet Active Media Technology and Information Processing (ICCWAMTIP). Piscataway, NJ, USA: IEEE. 238–244.

[48] Luceri A Bajwa MN Braun SA Malik MI Dengel A Ahmed S . ExAID: A multimodal explanation framework for computer-aided diagnosis of skin lesions. Comput Methods Programs Biomedicine. (2022) 215:106620. doi: 10.1016/j.cmpb.2022.106620, PMID: 35033756

[49] Zhao Y Barnaghi P Haddadi H . (2022). “ Multimodal federated learning on IoT data,” in Proceedings of the 2022 IEEE/ACM Seventh International Conference on Internet-of-Things Design and Implementation (IoTDI). Piscataway, NJ, USA: IEEE. 43–54.

[50] Qayyum A Ahmad K Ahsan MA Al-Fuqaha A Qadir J . Collaborative federated learning for healthcare: Multi-modal COVID-19 diagnosis at the edge. IEEE Open J Comput Soc. (2022) 3:172–84. doi: 10.1109/OJCS.2022.3206407, PMID: 25079929

[51] Ji J Yan D Mu Z . (2022). “ Personnel status detection model suitable for vertical federated learning structure,” in Proceedings of the 2022 6th International Conference on Machine Learning and Soft Computing. New York, NY, USA: ACM. 98–104.

[52] Chen Z Zhou H Xie Y Wang L Feng Q Yang W . Patient-level anatomy meets scanning-level physics: Personalized federated low-dose CT denoising empowered by large language model. IEEE Trans Med Imaging. (2025) 44:892–905. doi: 10.1109/CVPR52729.2023.01956, PMID: 25079929

[53] Yang Z Xia W Lu Z Chen Y Li X Zhang Y . Hypernetwork-based physics-driven personalized federated learning for CT imaging. IEEE Trans Neural Networks Learn Syst. (2023) 36:3136–50. doi: 10.1109/TNNLS.2023.3338867, PMID: 38100342

[54] Sumaiya N Ali A . (2024). Federated learning assisted deep learning methods fostered skin cancer detection: a survey. Front Biomed Technol. 36:3136–3150. doi: 10.48550/arXiv.2206.03709

[55] Malik PK Bhatt H Sharma M . AI integration in healthcare systems—A review of the problems and potential associated with integrating AI in healthcare for disease detection and diagnosis. In: In AI in disease detection: Advancements and applications. Wiley-IEEE Press, Piscataway, NJ, USA (2025). p. 191–213.

[56] Sashidharavi A Kanimozhi S Sasidhar P Pulipati PK Sruthi E Prakash P . Efficient AI: A synergistic approach to skin disease classification through multi-dataset fusion and attention mechanisms. Biomed Signal Process Control. (2025) 100:107141. doi: 10.1038/s41598-024-77196-x, PMID: 39478132 PMC11526108

[57] Palit R Gupta A Mandrekar D . Driving medical diagnostics forward: The role of AI in innovation and implementation. Cuestiones Fisioterapia. (2025) 54:155–84. doi: 10.3390/diagnostics13091532, PMID: 37174925 PMC10177193

[58] Kurtansky NR D’Alessandro BM Gillis MC Betz-Stablein B Cremara SE Garcia R . The SLICE-3D dataset: 400,000 skin lesion image crops extracted from 3D TBP for skin cancer detection. Sci Data. (2024) 11:884. doi: 10.1038/s41597-024-03743-w, PMID: 39143096 PMC11324883

[59] Borazjani K Khosravan N Ying L Hosseinalipour S . Multi-modal federated learning for cancer staging over non-IID datasets with unbalanced modalities. IEEE Trans Med Imaging. (2024) 44:556–73. doi: 10.1109/TMI.2024.3450855, PMID: 39196746

[60] Alhafiz FS Bashuhail AA . (2024). Non-IID medical imaging data on COVID-19 in the federated learning framework: impact and directions. COVID. 4(12):1985–2016. doi: 10.3390/covid4120140. PMID: 30654563

[61] Qin Z Yang H Wang Q Han Y Hu Q . (2023). Reliable and interpretable personalized federated learning, in: In Proceedings of the IEEE/CVF Conference on Computer Vision and Pattern Recognition, Piscataway, NJ, USA: IEEE. pp. 20422–31.

[62] Yan Y Wang H Huang Y He N Zhu L Xu Y . (2024). Cross-modal vertical federated learning for MRI reconstruction. IEEE J Biomed Health Inform. 28:6384–94. doi: 10.1109/JBHI.2024.3360720. Piscataway, NJ, USA: IEEE. PMID: 38294925

[63] Dalmaz O Mirza MU Elmas O Ozbey M Dar SU Ceylan E . One model to unite them all: Personalized federated learning of multi-contrast MRI synthesis. Med Image Anal. (2024) 94:103121. doi: 10.1016/j.media.2024.103121, PMID: 38402791

[64] Arangasamy R Kodela V Soumya G Srivastava S Meghashree AC . Federated attention-encrypted learning for privacy-preserving medical image classification. In: Proceedings of the 5th International Conference on Mobile Networks and Wireless Communications (ICMNWC) IEEE. (2025) 1–6.

[65] Khan R Taj SMA Noor A Zhu H Khan J Khan SU . Advanced federated ensemble internet of learning approach for cloud based medical healthcare monitoring system. Sci Rep. (2024) 14:26068. doi: 10.1038/s41598-024-77196-x, PMID: 39478132 PMC11526108

[66] Nazir S Kaleem M . Federated learning for medical image analysis with deep neural networks. Diagnostics. (2023) 13:1532. doi: 10.3390/diagnostics13091532, PMID: 37174925 PMC10177193

[67] Naeem A Anees T Naqvi RA Loh WK . A comprehensive analysis of recent deep and federated-learning-based methodologies for brain tumor diagnosis. J Personalized Med. (2022) 12:275. doi: 10.3390/jpm12020275, PMID: 35207763 PMC8880689

[68] Naz NS Mehmood MH Ahmed F Ahmad M Rehman AU Ismael WM . Privacy preserving skin cancer diagnosis through federated deep learning and explainable AI. Sci Rep. (2025) 15:36094. doi: 10.1038/s41598-025-19905-8, PMID: 41093964 PMC12528741

[69] Alhafiz FS Bashuhail AA . Non-IID medical imaging data on COVID-19 in the federated learning framework: impact and directions. COVID. (2024) 4:1985–2016. doi: 10.3390/covid4120140, PMID: 30654563

[70] Al-Rakhami MS AlQahtani SA Alawwad A . Effective skin cancer diagnosis through federated learning and deep convolutional neural networks. Appl Artif Intell. (2024) 38:2364145. doi: 10.1080/08839514.2024.2364145, PMID: 37339054

[71] Wang Y Wang Y Cai J Lee TK Miao C Wang ZJ . SSD-KD: A self-supervised diverse knowledge distillation method for lightweight skin lesion classification using dermoscopic images. Med Image Anal. (2023) 84:102693. doi: 10.1016/j.media.2022.102693, PMID: 36462373

[72] Adepu AK Sahayam S Jayaraman U Arramraju R . Melanoma classification from dermatoscopic images using knowledge distillation for highly imbalanced data. Comput Biol Med. (2023) 154:106571. doi: 10.1016/j.compbiomed.2023.106571, PMID: 36709518

[73] Wang Y Cai J Louie DC Wang ZJ Lee TK . Incorporating clinical knowledge with constrained classifier chain into a multimodal deep network for melanoma detection. Comput Biol Med. (2021) 137:104812. doi: 10.1016/j.compbiomed.2021.104812, PMID: 34507158

[74] El Mrabet A Benaly M Alihamidi I Kouach B Hloul L El Gouri R . Enhancing early detection of skin cancer in clinical practice with hybrid deep learning models. Engineering Technol Appl Sci Res. (2025) 15:20927–33. doi: 10.48084/etasr.9753

[75] Hashmani MA Jameel SM Rizvi SSH Shukla S . An adaptive federated machine learning-based intelligent system for skin disease detection: A step toward an intelligent dermoscopy device. Appl Sci. (2021) 11:2145. doi: 10.3390/app11052145, PMID: 30654563

